# Telomere length associates with chronological age and mortality across racially diverse pulmonary fibrosis cohorts

**DOI:** 10.1038/s41467-023-37193-6

**Published:** 2023-03-17

**Authors:** Ayodeji Adegunsoye, Chad A. Newton, Justin M. Oldham, Brett Ley, Cathryn T. Lee, Angela L. Linderholm, Jonathan H. Chung, Nicole Garcia, Da Zhang, Rekha Vij, Robert Guzy, Renea Jablonski, Remzi Bag, Rebecca S. Voogt, Shwu-Fan Ma, Anne I. Sperling, Ganesh Raghu, Fernando J. Martinez, Mary E. Strek, Paul J. Wolters, Christine Kim Garcia, Brandon L. Pierce, Imre Noth

**Affiliations:** 1 Section of Pulmonary & Critical Care, Department of Medicine, The University of Chicago, Chicago, IL USA; 2 Committee on Genetics, Genomics and Systems Biology, The University of Chicago, Chicago, IL USA; 3 Division of Pulmonary & Critical Care Medicine, Department of Internal Medicine, University of Texas Southwestern, Dallas, TX USA; 4 Division of Pulmonary, Critical Care & Sleep Medicine, Department of Medicine, University of California, Davis, Sacramento, CA USA; 5 Section of Pulmonary & Critical Care Medicine, Department of Medicine, University of California, San Francisco, CA USA; 6 Department of Radiology, The University of Chicago, Chicago, IL USA; 7 Division of Pulmonary, Allergy and Critical Care Medicine, Columbia University Medical Center, New York, NY USA; 8 Division of Pulmonary & Critical Care Medicine, Department of Medicine, University of Virginia, Charlottesville, VA USA; 9 Division of Pulmonary, Critical Care and Sleep Medicine, University of Washington Medical Center, Seattle, WA USA; 10 Pulmonary Critical Care Medicine, Weill Cornell Medicine, New York City, NY USA; 11 Department of Public Health Sciences, The University of Chicago, Chicago, IL USA; 12 Department of Human Genetics, The University of Chicago, Chicago, IL USA

**Keywords:** Respiratory tract diseases, Telomeres, Geriatrics

## Abstract

Pulmonary fibrosis (PF) is characterized by profound scarring and poor survival. We investigated the association of leukocyte telomere length (LTL) with chronological age and mortality across racially diverse PF cohorts. LTL measurements among participants with PF stratified by race/ethnicity were assessed in relation to age and all-cause mortality, and compared to controls. Generalized linear models were used to evaluate the age-LTL relationship, Cox proportional hazards models were used for hazard ratio estimation, and the Cochran–Armitage test was used to assess quartiles of LTL. Standardized LTL shortened with increasing chronological age; this association in controls was strengthened in PF (R = −0.28; *P* < 0.0001). In PF, age- and sex-adjusted LTL below the median consistently predicted worse mortality across all racial groups (White, HR = 2.21, 95% CI = 1.79–2.72; Black, HR = 2.22, 95% CI = 1.05–4.66; Hispanic, HR = 3.40, 95% CI = 1.88–6.14; and Asian, HR = 2.11, 95% CI = 0.55–8.23). LTL associates uniformly with chronological age and is a biomarker predictive of mortality in PF across racial groups.

## Abstract (web summary)

The association of telomere length with age and mortality across racially diverse pulmonary fibrosis populations is unknown. Here, the authors show that leukocyte telomere length associates with chronologic age and is predictive of mortality in pulmonary fibrosis across racial groups.

## Introduction

Pulmonary fibrosis (PF) is a complex group of lung diseases characterized by scarring in the interstitium of the lung; median survival in its most common subtype, idiopathic PF (IPF), is only 3–5years^1–3^. While numerous investigations over the past decade have illuminated the pathophysiology of PF, much remains unknown about the accuracy of current prognostic indices across individuals from different racial or ethnic groups. An improved understanding of the prognostic value of PF biomarkers across racial and ethnic groups would enhance pharmacotherapeutic precision and inform targeted management within the spectrum of diagnoses among patients of diverse backgrounds.

Recently discovered genomic biomarkers in the peripheral blood represent a promising avenue toward achieving precision medicine in patients with PF^4^. Short leukocyte telomere length (LTL), a marker of cellular senescence, is associated with germline mutations in rare and common gene variants involved in telomere biology and linked to PF incidence as well as mortality^5–9^. LTL expectantly shortens with increasing age, but PF patients are disproportionately enriched for short LTL^10–12^. However, the current understanding of the impact of genetic and genomic biomarkers on patient outcomes in PF is confounded by the lack of inclusion of racial and ethnic minorities in most genetic studies to date^13^. In particular, studies linking LTL to clinical outcomes in PF have not been performed in racially diverse cohorts. Due to these shortcomings, the optimal prognostic value of results derived from these genomic studies is unknown in diverse PF populations, as is whether telomere shortening is uniformly associated with increasing age in PF across racial/ethnic groups.

The primary objective of this study was to comparatively analyze differences in LTL between racial/ethnic groups within PF and within control populations. The secondary objectives were (a) to investigate whether LTL is associated with chronological age across racially and ethnically diverse PF cohorts and (b) to assess the relationship between LTL and mortality risk across ethnic groups with PF. We hypothesized that shorter telomere length is associated with increased age and greater mortality across diverse racial groups, underscoring the importance of this biomarker in all patients with PF.

## Results

A total of 7854 individuals were included in the study, 2046 participants with PF and 5808 HRS control participants. The number of participants with PF and control individuals analyzed across the different racial/ethnic groups is presented in Table 1 (characteristics of participants with PF stratified by enrollment site can be found in Supplementary Table 1 in the Supplement). The mean ± SD age was 65 ± 11 years for PF participants, and 69 ± 10 years for HRS controls. Black participants had the youngest mean age across all sites, and age at PF diagnosis was lower than in White participants (CHICAGO 54 vs. 65 years; *P* < 0.001; CALIFORNIA 60 vs. 68 years; *P* < 0.001; TEXAS 53 vs. 64 years; *P* < 0.001; IPFNet 61 vs. 67 years; *P* = 0.25; Supplementary Fig. 1). Male participants were 54% of those with PF and 41% of HRS controls. The mean BMI was high in participants with PF (28 ± 6 kg/m^2^) and control participants (29 ± 6 kg/m^2^). Tobacco use was common in PF (58%) and control participants (57%). The median follow-up time for PF participants was 31 (IQR 14–60) months.

**Table 1:** Pulmonary fibrosis (PF) and health retirement survey (HRS) cohorts stratified by racial ancestry

Characteristics^a^	White	Black	Hispanic	Asian	*P* value^#^
PF (*n* = 2046)	(*n* = 1613)	(*n* = 162)	(*n* = 187)	(*n* = 70)	
Age, years	66.0 (10.4)	54.9 (12.2)	59.7 (12.8)	65.7 (12.9)	<0.001
Male	918 (56.9)	44 (27.2)	90 (48.1)	39 (55.7)	<0.001
Ever Smoker	978 (60.8)	70 (43.2)	96 (51.3)	26 (37.7)	<0.001
Body Mass Index	29.2 (5.7)	29.0 (6.5)	29.8 (6.7)	25.9 (5.1)	0.001
Lung function					
FVC (% predicted)	68.4 (19.1)	59.4 (18.4)	62.0 (19.2)	66.7 (18.2)	<0.001
FEV_1_ (% predicted)	74.1 (19.7)	62.9 (20.5)	65.7 (19.6)	74.4 (25.0)	<0.001
DL_CO_ (% predicted)	48.4 (19.2)	41.8 (18.9)	45.0 (17.3)	45.3 (17.1)	<0.001
PF sub-category					
IPF	653 (40.5)	13 (8.0)	49 (26.2)	20 (28.6)	<0.001
IPAF	153 (9.5)	30 (18.5)	15 (8.0)	8 (11.4)	0.006
CTD-ILD	200 (12.4)	89 (54.9)	46 (24.6)	14 (20.0)	<0.001
FHP	359 (22.3)	14 (8.6)	34 (18.2)	11 (15.7)	0.001
Unclassifiable/others	248 (15.4)	16 (9.9)	43 (23.0)	17 (24.3)	<0.001
HRS (*n* = 5808)	(*n* = 4319)	(*n* = 779)	(*n* = 614)	(*n* = 96)	
Age, years	70.2 (10.2)	67.8 (9.9)	66.0 (10.5)	65.5 (11.0)	<0.001
Male	1817 (42.1)	280 (35.9)	235 (38.3)	39 (40.6)	0.007
Ever smoker	2493 (58.0)	442 (57.1)	320 (52.7)	40 (41.7)	0.001
Body mass index	27.9 (5.7)	29.8 (6.6)	29.0 (5.6)	26.7 (5.9)	<0.001

^a^Categorical variables presented as *n* (%); continuous variables presented as means (SD).

^#^*P* value for chi-squared (categorical data) or one-way ANOVA (continuous data) comparing all four main racial groups. Patients with mixed or other racial ancestry not depicted above, *n* = 14. Exception for participants in PF cohort; smoking status, *n* = 2041; Body mass index = 1505; *FVC* forced vital capacity, *n* = 2015; *FEV1* forced expiratory volume in the first second, *n* = 1254; *DL*_CO_ diffusing capacity of the lungs, *n* = 1959. *ILD* interstitial lung disease; *IPF* idiopathic pulmonary fibrosis, *n* = 735, *IPAF* interstitial pneumonia with autoimmune features, *n* = 207; *CTD-ILD* connective tissue disease associated-ILD, *n* = 349; *FHP* fibrotic hypersensitivity pneumonitis, *n* = 422; unclassifiable/other ILD, *n* = 333.

### Telomere length across racial/ethnic groups

**Fig. 1:**
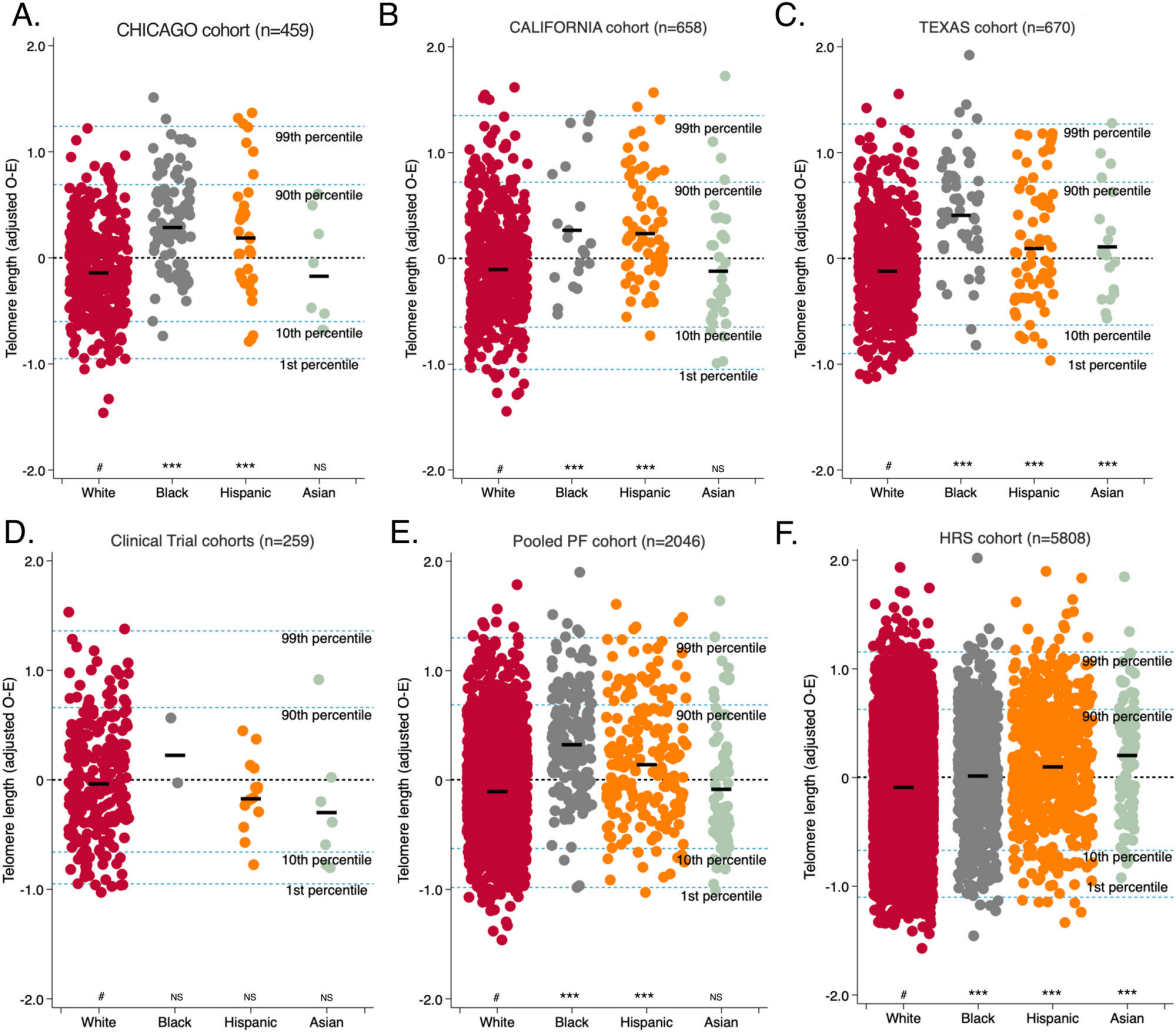
Mean observed minus expected (O–E; age and gender-adjusted) leukocyte telomere length (TL) is longest in Black subjects with pulmonary fibrosis (PF). Study cohort stratified according to White(W), Black(Bl), Hispanic(H), and Asian(As); **A** CHICAGO cohort, (W, *n* = 332; Bl, *n* = 84; H, *n* = 35; and As, *n* = 8); mean TL (relative T/S): W, Bl, H, and As = 1.38(SD 0.27), 1.62(SD 0.23), 1.51(SD 0.34), and 1.48(0.21), which correspond to mean age and gender-adjusted TL(O–E) of −0.09(0.43), 0.34(0.46), 0.24(0.59), and −0.13(0.53), respectively. **B** CALIFORNIA cohort, (W, *n* = 499; Bl, *n* = 21; H, *n* = 70; and As, *n* = 36); mean TL, base pairs(bp): W, Bl, H, and As = 6058bp(SD 650), 6267bp(SD 679), 6206bp(SD 662), and 6255bp(SD 682), which correspond to mean age and gender-adjusted TL(O–E) of −0.06(0.49), 0.32(0.56), 0.28(0.49), and −0.08(0.58), respectively. **C** TEXAS cohort, (W, *n* = 527; Bl, *n* = 55; H, *n* = 68; and As, *n* = 19); mean TL (relative T/S): W, Bl, H, and As = 1.29(SD 0.28), 1.50(SD 0.26), 1.41(SD 0.29) and 1.37(0.34), which correspond to mean age and gender-adjusted TL(O–E) of −0.07(0.46), 0.46(0.51), 0.14(0.57), and 0.16(0.55), respectively. **D** IPFNet cohort, (W, *n* = 236; Bl, *n* = 2; H, *n* = 14; and As, *n* = 7); mean TL(relative T/S): W, Bl, H, and As = 1.11(SD 0.21), 1.35(SD 0.43), 1.17(SD 0.23), and 1.19(0.18), which correspond to mean age and gender-adjusted TL(O–E) of 0.013(SD 0.50), 0.27(SD 0.41), −0.13(SD 0.33), and −0.25 (0.60), respectively. **E** Pooled PF cohort, (W, *n* = 1611; Bl, *n* = 162; H, *n* = 183; and As, *n* = 70); mean age and gender-adjusted TL(O–E) of −0.06(0.47), 0.37(0.49), 0.19(0.54), and −0.04(0.57), respectively. **F** HRS cohort, (W, *n* = 4319; Bl, *n* = 779; H, *n* = 614; and As, *n* = 96); mean TL(relative T/S) W, Bl, H, and As = 1.34(SD 0.65), 1.57(SD 1.11), 1.41 (SD 0.59), and 1.39(0.63), which correspond to mean age and gender-adjusted TL(O–E) of −0.04(0.49), 0.08(0.48), 0.16(0.51), and 0.19(0.53), respectively. Thick short black lines show the median for each subgroup. The black dotted line shows the median TL for each cohort group; the blue dotted lines show approximate age-adjusted prediction bands in percentiles for each cohort. Group comparisons between white subjects (#) and other racial subgroups were conducted using the student’s *T*-test; ***P < 0.0005; NS not significant (*P* ≥ 0.05).

Among participants with PF, LTL differed across racial groups and was longest in Black subjects. The mean standardized LTL at PF diagnosis was longer in Black participants 0.37 ± 0.49 than in White participants −0.06 ± 0.47 in the pooled PF cohort (*P* < 0.0005), and when stratified by participant recruitment site. Among HRS control participants, mean standardized LTL was also longer in Black participants 0.08 ± 0.48 compared to White participants −0.04 ± 0.49 (*P* < 0.0005). The magnitude of this difference in LTL across racial groups was 3.6-fold larger in the PF cohort than in controls (Fig. 1, Supplementary Table 2). Among the control cohort, the odds of having LTL below the median was lower in Black, Hispanic, and Asian subjects (OR = 0.7, 95% CI = 0.6–0.8, *P* < 0.001; OR = 0.5, 95% CI = 0.5–0.6, *P* < 0.001; and OR = 0.6, 95% CI = 0.4–0.9, *P* = 0.015, respectively) when compared to White subjects. Similarly, among the PF cohort, the odds of having LTL below the median was lower in Black and Hispanic subjects (OR = 0.2, 95% CI = 0.2–0.4, *P* < 0.001; and OR = 0.5, 95% CI = 0.4–0.7, *P* < 0.001, respectively) but not different in Asian subjects (OR = 0.9, 95% CI = 0.5–1.4, *P* = 0.55) when compared to White subjects (Fig. 1). The difference in mean LTL between Black and White subjects with PF was diminished in propensity-score matched analyses (0.37 ± 0.49 vs. 0.33 ± 0.56, respectively) (Supplementary Fig. 2). When assessing quartiles of standardized LTL stratified by race/ethnicity, the mean LTL uniformly increased from the lowest quartile (Q1) to the highest quartile (Q4) across all racial groups(Supplementary Table 3).

### Sex, disease subtypes, and telomere length

The mean unadjusted LTL was consistently shorter in males than females across all PF cohorts and HRS control participants(Supplementary Table 4). This sex disparity was also uniformly observed across all racial/ethnic groups assessed(Supplementary Table 4).

**Fig. 2:**
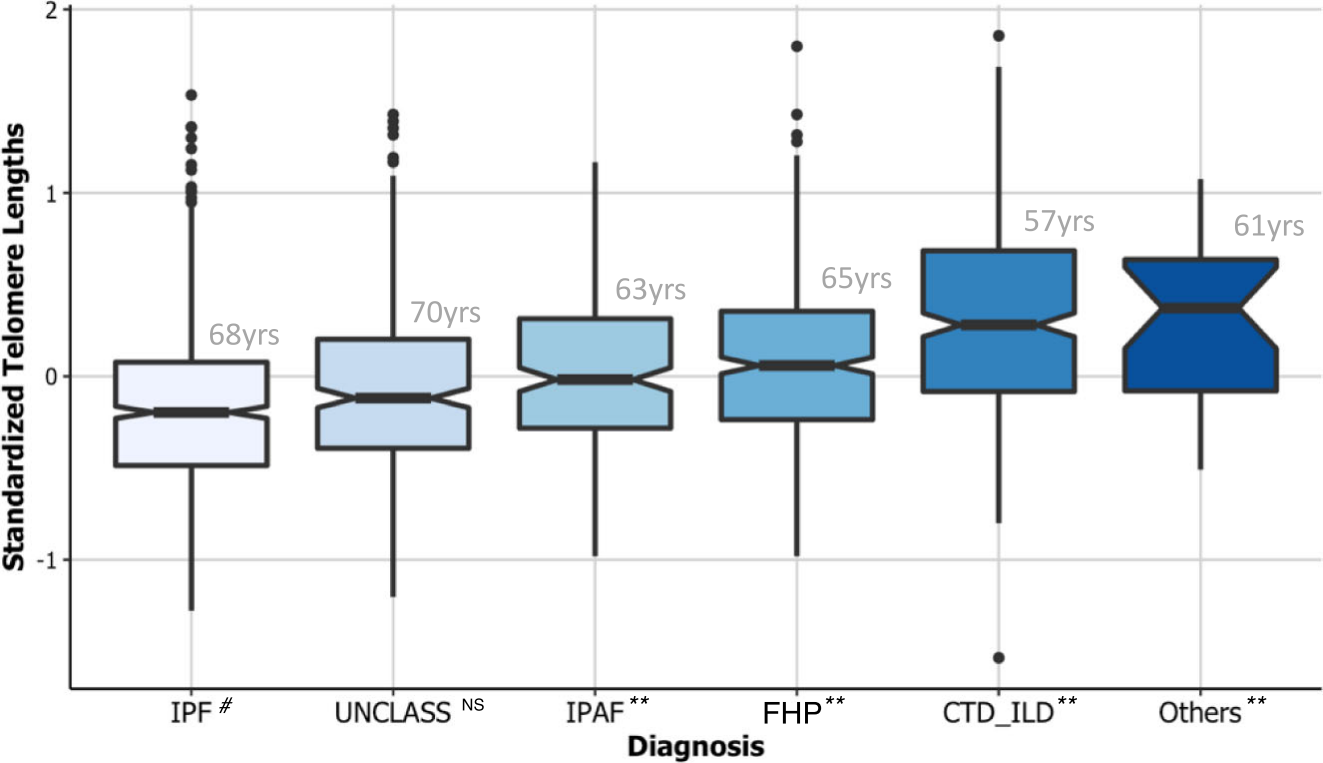
Median standardized leukocyte telomere length is shortest in subjects with idiopathic PF and longest in subjects with connective tissue disease-related interstitial lung disease (CTD-ILD), and other ILDs. Patients with pulmonary fibrosis (PF) stratified by diagnostic subgroup and median age of subgroup (gray); idiopathic pulmonary fibrosis (IPF, *n* = 735), unclassifiable (UNCLASS, *n* = 260), interstitial pneumonia with autoimmune features (IPAF, *n* = 207), fibrotic hypersensitivity pneumonitis (FHP, *n* = 422), CTD-ILD, *n* = 349, and other ILDs (Others, *n* = 73). All telomere lengths depicted are observed minus expected (O–E; age and gender-adjusted). The notched colored box shows the interquartile range (25th to 75th percentile), the horizontal thick black line indicates the median, the vertical upper and lower whiskers represent values outside the middle 50% (the upper 25% of values and the lower 25% of values, respectively), the whisker boundaries represent the maximum and minimum values, and the black dots represent outlier values. Boxplot notch displays the confidence interval around the median based on the median ± 1.58 × IQR/sqrt(*n*). Group comparisons between IPF (#) and other diagnostic subgroups were conducted using Mood’s median test; **P < 0.0005; NS not significant (*P*  ≥ 0.05).

Subtypes of PF differed in their baseline demographics and in their standardized LTL measurements after adjusting for age and sex (Supplementary Table 5). Subjects with IPF were older (median age 68 years) and had the shortest median LTL −0.20 (IQR −0.49–0.08) (Fig. 2). Comparatively, subjects with interstitial pneumonia with autoimmune features (median age 63 years; median LTL −0.02, IQR −0.29–0.32), fibrotic hypersensitivity pneumonitis (median age 65 years; median LTL 0.06, IQR −0.24–0.36), connective tissue disease-related ILD (median age 57 years; median LTL 0.28, IQR −0.08–0.69), and other ILDs (median age 61 years; median LTL 0.06, IQR −0.17–0.47) were younger and had longer LTL (*P* < 0.0005). Observed LTL measurements for participants with the unclassifiable PF subtype (median age 70 years; median LTL −0.13, IQR −0.42–0.17) did not differ from those with IPF (*P* = 0.20) (Fig. 2). Across PF subtypes, White subjects had the shortest LTL (Supplementary Tables 6–8).

### Telomere length and chronological age

**Fig. 3:**
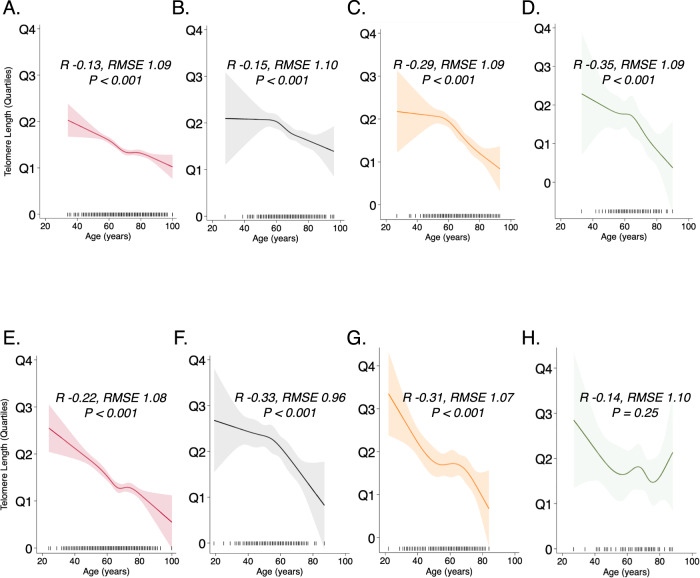
Correlation of leukocyte telomere length (TL) with age. TL demonstrates a nonlinear negative correlation with age across diverse racial groups in healthy subjects **A** Whites; **B** Blacks; **C** Hispanics; and **D** Asians; that is altered in subjects with pulmonary fibrosis **E** Whites; **F** Blacks; **G** Hispanics; and **H** Asians. Statistical test: Restricted cubic spline regression of TL on age across racial/ethnic groups. Model goodness of fit indicated by R (correlation coefficient), RMSE (root mean square error) of restricted cubic spline (solid line), and *P* value reported for the respective populations. Lighter colored band indicates 95% confidence intervals; vertical bars on the horizontal scale indicate individual observations.

**Fig. 4:**
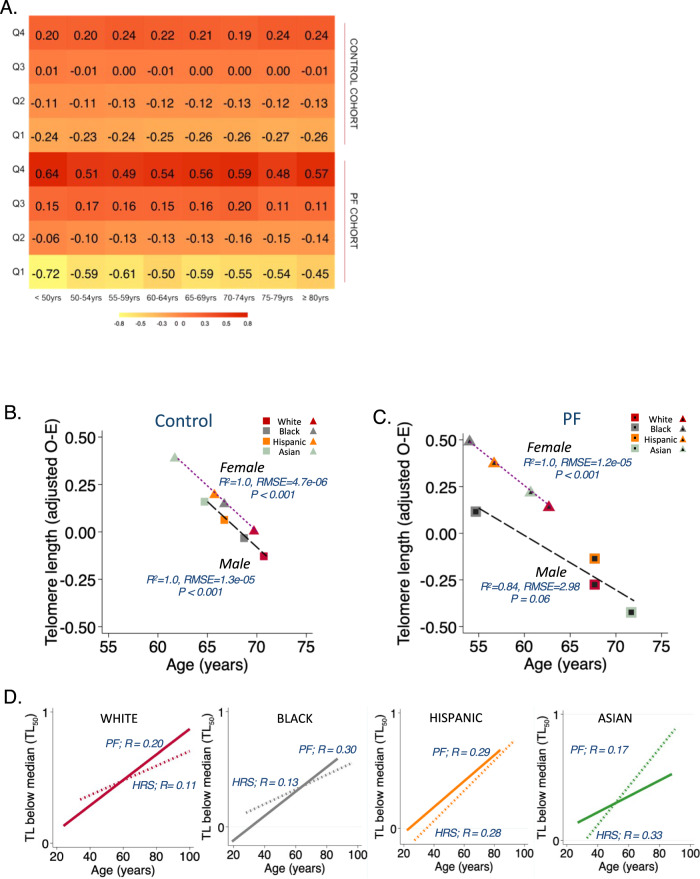
Standardized leukocyte telomere length (TL) measured by qPCR demonstrates wider interquartile range variation in pulmonary fibrosis (PF) and decreases with increasing age. **A** Age-stratified mean values of TL measurements increase within quartiles (Q) from the first quartile (Q1) to the fourth quartile (Q4) for 2046 subjects with PF and 5808 control subjects. Median TL for each race (White *red*, Black gray, Hispanic orange, and Asian green) stratified by sex (male = squares, female = triangles) among the **B** Control population (HRS); and **C** PF population); statistical test: generalized linear regression of sex-stratified median TL across racial/ethnic groups. *R*² (the coefficient of determination), RMSE (root mean squared error), and *P* value reported for each subgroup. **D** Shorter TL below the median (TL_50_) has greater prevalence with increasing age across all racial groups in the HRS control population (dashed lines), but this association was stronger in White and Black subjects with PF (solid lines). Subjects with PF (White *n* = 1613, Black, *n* = 162, Hispanic, *n* = 187, Asian, *n* = 70). Control subjects (White *n* = 4319, Black, *n* = 779, Hispanic, *n* = 614, Asian, *n* = 96). Other racial groups [*n* = 14] are not included in the graphs above. TL depicted are observed minus expected (O–E; age and gender-adjusted). Purple dotted line = fitted values for females and black dashed line = fitted values for males.

With increasing age, the unadjusted LTL decreased in participants with PF at all recruitment sites and among HRS control subjects (Supplementary Tables 7–9). Chronological age had a negative correlation with LTL across diverse racial groups in HRS control subjects and PF participants (Fig. 3). Assessment of LTL quartiles showed an expected increase in mean LTL measurements from the first (Q1) to the fourth quartile (Q4) consistently across age categories stratified by 5-year epochs (Fig. 4A). However, participants with PF had wider IQR for all age categories than the HRS control participants (Fig. 4A).

Standardized LTL was negatively associated with chronological age across White, Black, Hispanic, and Asian HRS control participants (*R* = −0.13, 95% CI = −0.16 to −0.10; *R* = −0.15, 95% CI = −0.22 to −0.09; *R* = −0.29, 95% CI = −0.36 to −0.21; and *R* = −0.35, 95% CI = −0.51 to −0.16, respectively). This modest negative association was strengthened across White, Black, Hispanic, and Asian PF subjects (*R* = −0.22, 95% CI = −0.27 to −0.17; *R* = −0.33, 95% CI = −0.46 to −0.18; *R* = −0.31, 95% CI = −0.44 to −0.18; and *R* = −0.14, 95% CI = −0.36 to 0.10, respectively) where the highest correlation occurred among Black PF participants (Table 2 and Supplementary Table 9). Assessment of the median LTL within each substratum of race and sex, showed a strong correlation across racial/ethnic groups of the age-telomere length relationship for male and female HRS control subjects (*R*^*2*^ = 1.0; *P* < 0.001) (Fig. 4B). PF disrupted this uniform linear relationship across racial/ethnic groups with a non-significant correlation among male PF participants (*R*^*2*^ = 0.84; *P* = 0.06), and LTL was shortest in older White or Asian males with PF (Fig. 4C). Among controls, Hispanic ethnicity significantly impacted the age-LTL relationship (interaction term *P* = 0.027) while race did not. In PF, Hispanic ethnicity, White, and Black race significantly impacted the age-LTL relationship (interaction term *P* < 0.001) while Asian race did not(interaction term *P* = 0.25). In an assessment of the overall age/LTL relationship between PF and HRS control subjects across racial/ethnic categories, our model shows that PF exerts an independent effect on this age/LTL relationship within the different racial/ethnic categories with significant interaction *P* values (Supplementary Table 7).

**Table 2:** Models depicting the association of leukocyte telomere length (LTL) with age and mortality across diverse racial populations

Characteristics	White	Black	Hispanic	Asian	Combined
Regression models for Age^*^					
Unadjusted LTL					
PF Cohort	(*n* = 1613)	(*n* = 162)	(*n* = 187)	(*n* = 70)	(*n* = 2046)
All, *R* (Root MSE)	−0.21 (0.27)	−0.29 (0.24)	−0.39 (0.28)	−0.17 (0.29)	−0.30 (0.28)
95% CI	−0.26 to −0.17	−0.43 to −0.14	−0.51 to −0.26	−0.40 to 0.07^+^	−0.33 to −0.26
HRS Cohort	(*n* = 4319)	(*n* = 779)	(*n* = 614)	(*n* = 96)	(*n* = 5808)
All, *R* (Root MSE)	−0.04 (0.65)	−0.02 (1.11)	−0.12 (0.59)	−0.07 (0.63)	−0.05 (0.73)
95% CI	−0.07 to −0.01	−0.10 to −0.05	−0.42 to −0.05	−0.26 to −0.14	−0.08 to −0.03
Standardized LTL^**^					
PF cohort	(*n* = *1613)*	(*n* = 162)	(*n* = 187)	(*n* = 70)	(*n* = 2046)
All, *R* (Root MSE)	−0.22 (1.08)	−0.33 (0.96)	−0.31 (1.07)	−0.14 (1.10)	−0.28 (1.08)
95% CI	−0.27 to −0.17	−0.46 to −0.18	−0.44 to −0.18	−0.36 to 0.10^+^	−0.32 to −0.24
HRS cohort	(*n* = 4319)	(*n* = 779)	(*n* = 614)	(*n* = 96)	(*n* = 5808)
All, *R* (Root MSE)	−0.13 (1.09)	−0.15 (1.10)	−0.29 (1.09)	−0.35 (1.09)	−0.17 (1.10)
95% CI	−0.16 to −0.10	−0.22 to −0.09	−0.36 to −0.21	−0.51 to −0.16	−0.19 to −0.14
PF mortality risk models	(*n* = 1613)	(*n* = 162)	(*n* = 187)	(*n* = 70)	(*n* = 2046)
^#^Overall crude mortality rate	8.92	5.27	9.62	6.62	8.49
(95% CI)	(8.16–9.76)	(3.91–7.10)	(7.47–12.38)	(3.84–11.40)	(7.83–9.20)
^+^Mortality incidence rate ratio, TL_50_	2.10	2.09	2.79	2.67	2.31
(95% CI)	(1.71–2.59)	(1.07–4.07)	(1.66–4.69)	(0.89–8.03)	(1.93–2.78)
*Mortality hazard ratio for TL_50_	2.21	2.22	3.40	2.11	2.47
(95% CI)	(1.79–2.72)	(1.05–4.66)	(1.88–6.14)	(0.54–8.23)	(2.05–2.97)

*R* Pearson’s bivariate correlation coefficient. *Root MSE* root mean squared error. *PF* pulmonary fibrosis. *HRS* health retirement survey.

*Hazard Ratio for TL50 in multivariable Cox regression models adjusting for forced vital capacity, diffusing capacity of the lungs for carbon monoxide, interstitial lung disease subtype, and hospital center. For multivariable models, White *n* = 1534, Black *n* = 145, Hispanic *n* = 175, Asian *n* = 64, All (including patients with race categorized as other, *n* = 14) *n* = 1932.*P <  0.001 for all regression models except where denoted by +. **Standardized telomere lengths in quartiles.

^#^Overall crude mortality rate computed per 100 person-yrs. ^#^*P* value for Mantel–Haenszel test statistic.

^+^Mortality incidence rate ratios in subjects with age- and gender-adjusted leukocyte telomere length below the median (TL_50_) were estimated using a generalized linear model with a Poisson distribution and logistic regression link adjusting for age, gender, forced vital capacity, diffusing capacity of the lungs for carbon monoxide, and hospital center.

We analyzed the association between LTL and chronological age using age- and gender-adjusted LTL below the median (TL_50_). The association of LTL with chronological age remained modest with the use of this binary threshold across all racial/ethnic subgroups of the HRS population (*R* = 0.11–0.33) and among participants with PF(*R* = 0.17–0.30) (Table 2 and Fig. 4D).

**Fig. 5:**
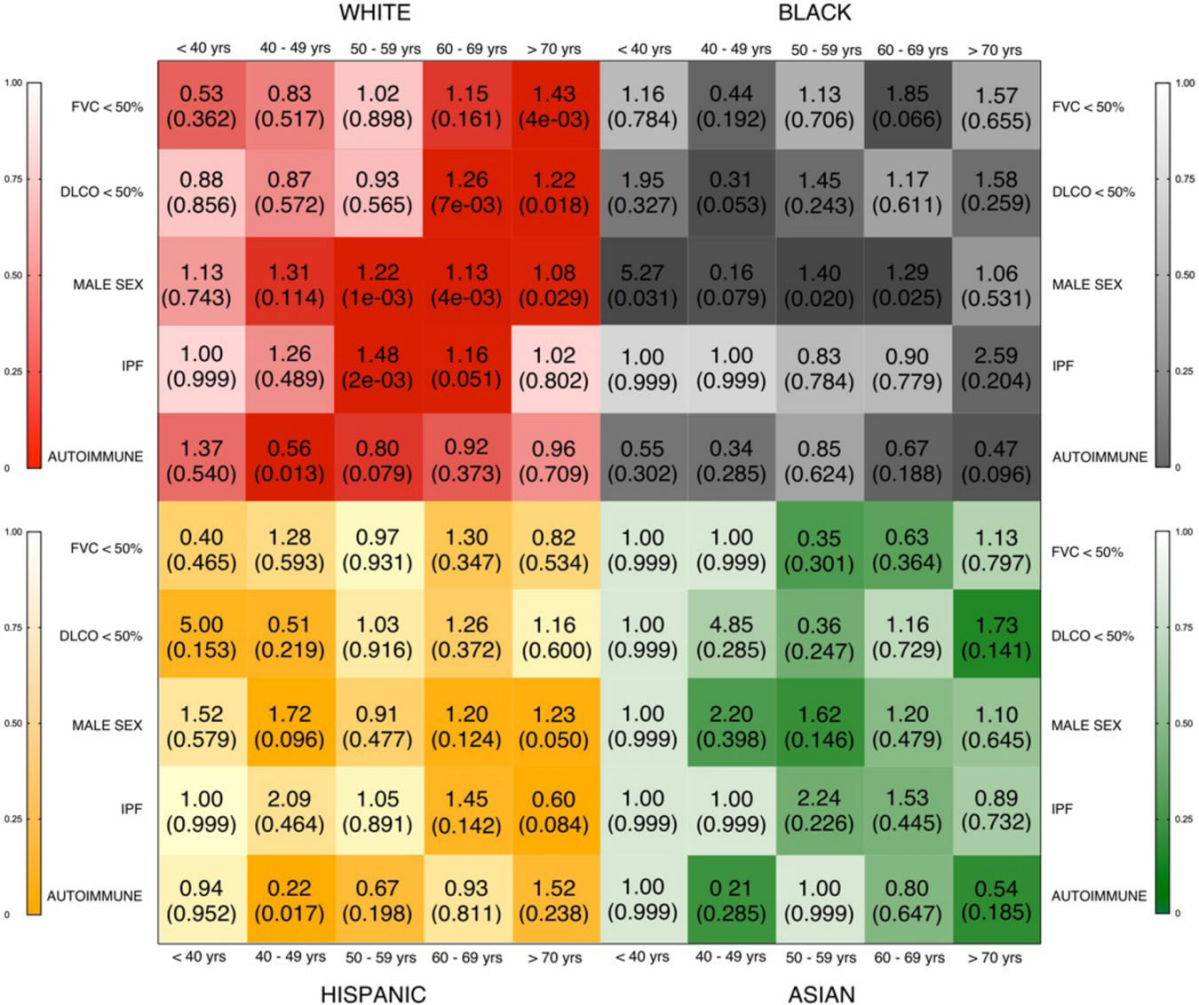
Racial differences in age-specific odds ratio (OR) for clinical predictors of respiratory impairment (y-axis) per quartile decrease in leukocyte telomere length (TL). Data stratified by race/ethnicity (panels) and age group (x-axis) at diagnosis of pulmonary fibrosis. OR and *P* value (in parenthesis) depicted for White subjects—top left panel (red), Black subjects—top right panel (gray), Hispanic subjects—bottom left panel (orange), and Asian subjects—bottom right panel (green). Binomial logistic regression models were used to compute OR and to determine the significance of association (*P* < 0.05) of TL with clinical variables. OR displayed within the corresponding box (OR on top and *P* value in parentheses) and the adjacent bar scales the statistical significance of the odds likelihood. The color of each box represents the magnitude of the statistical significance (darker color = greater statistical significance; lighter color = lesser statistical significance). FVC forced vital capacity below 50% predicted; DLCO diffusing capacity of the lung for carbon monoxide below 50% predicted; MALE SEX male, IPF idiopathic pulmonary fibrosis, AUTOIMMUNE autoimmune-related interstitial lung disease.

For each quartile decreases in LTL, the age-specific odds for developing predictors of respiratory impairment, including FVC and DLCO, differed across racial groups with PF (Fig. 5). In the sixth and seventh decades of life, decreasing LTL was associated with increased odds for IPF in White participants, and increased odds of being male in both Black and White participants.

### Telomere length and mortality

Among participants with PF, the absolute mortality rate and mortality incidence rate ratios for individuals with age- and gender-adjusted LTL below the median (TL_50_) were lowest among Black participants and highest among Hispanic participants (Table 2). When stratifying all racial/ethnic PF subgroups by the ILD-GAP score as an index of disease severity, LTL was observed to be shorter with increasing PF severity (Supplementary Table 10).

**Fig. 6:**
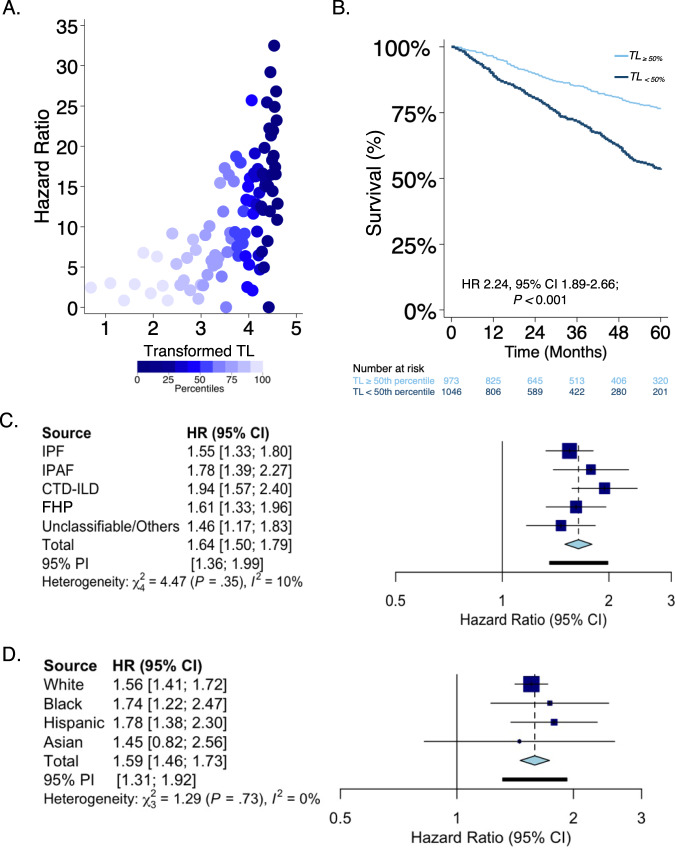
Shorter leukocyte telomere length (TL) consistently predicts worse survival patterns in pulmonary fibrosis (PF). **A** Scatter plot of mortality hazard ratios (HR)^*^ in PF by transformed TL (negative log-transformed inverse of one minus percentile TL) comparing each centile of TL to the highest TL centile. The plot depicts increasing mortality hazard with shorter TL. **B** Survival stratified by age and gender-adjusted TL below the median (TL_<50%_) vs. above the median (TL_≥50%_) in the PF cohort. Unadjusted Cox proportional hazard ratio (HR) and 95% confidence interval of this estimate are depicted with its respective *P* value. Fixed effect mortality hazard estimates for quartiles of leukocyte telomere length (TL_Q_) adjusted for age, gender, FVC, DLCO, ILD subtype, and hospital center categorized by **C** PF subtype, and **D** race/ethnicity. HR depicted per quartile increase in TL*.* FVC forced vital capacity, DLCO diffusing capacity of the lungs, ILD interstitial lung disease, IPF idiopathic pulmonary fibrosis, *n* = 735; IPAF interstitial pneumonia with autoimmune features, *n* = 207; CTD-ILD connective tissue disease associated-ILD, *n* = 349; FHP fibrotic hypersensitivity pneumonitis, *n* = 422; unclassifiable/other ILD, *n* = 333. White *n* = 1613, Black *n* = 162, Hispanic *n* = 187, Asian *n* = 70, others *n* = 14, All patients *n* = 2046. The navy-blue boxes within the forest plot represent the point estimate for the mortality hazard ratio for each cohort, the thin horizontal line represents its 95% confidence interval, the vertical line is the line of no effect, and the diamond represents the overall effect estimate.

**Fig. 7:**
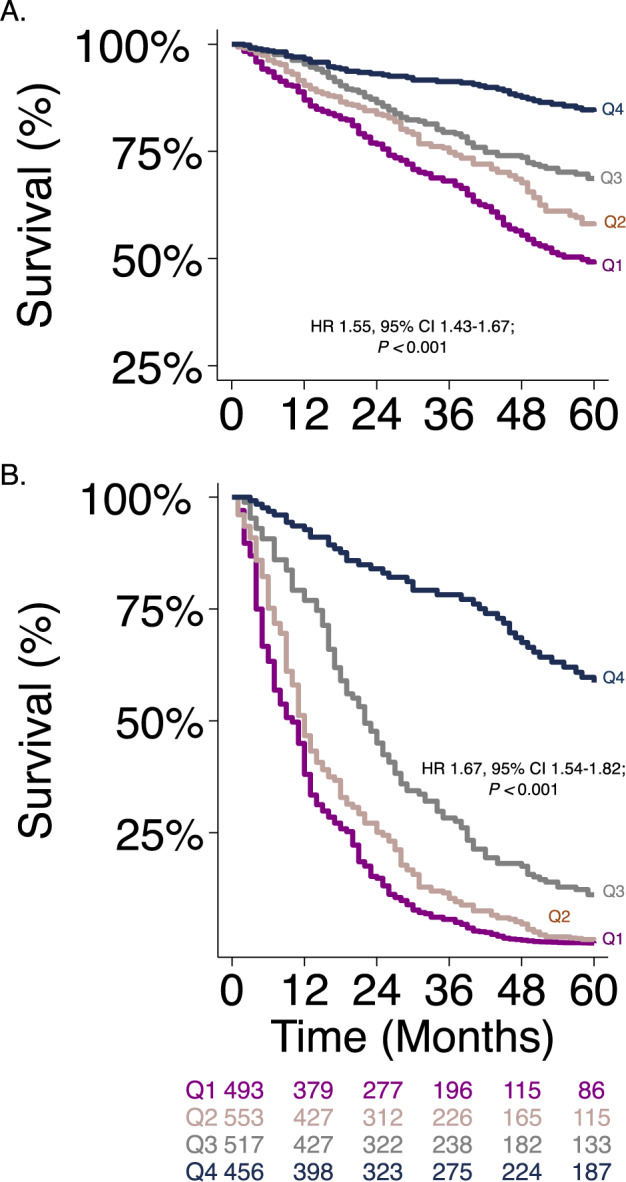
Shorter leukocyte telomere length consistently predicts worse survival. **A** Kaplan–Meier survival curve according to age and gender-adjusted leukocyte telomere length in quartiles (TL_Q_). **B** Kaplan–Meier survival curves according to TL_Q_ adjusted for age, gender, FVC, DLCO, ILD subtype, and hospital center. Cox proportional hazard ratio (HR) and 95% confidence interval of this estimate are depicted with its respective *P* value in each plot. FVC forced vital capacity, DLCO diffusing capacity of the lung for carbon monoxide, ILD interstitial lung disease.

In the pooled PF population, standardized LTL as a continuous measure was associated with mortality, and shorter LTL consistently predicted worsened mortality (Fig. 6A). When compared with participants who had LTL at or above the median (TL_≥50_), participants with TL_50_ had poorer survival across all study sites, across PF subtypes, and when substratified by racial/ethnic groups (Fig. 6B and Supplementary Figs. 3–5). These findings remained consistent even after adjusting for age, sex, and disease severity. Each quartile decrease in LTL was associated with a higher risk of death in participants with PF for each subtype of PF, and across the different racial/ethnic groups (Figs. 6C, D, 7).

Compared to subjects with TL_≥50_ in multivariable Cox proportional hazards models adjusted for age, sex, FVC, DLCO, PF subtype, and study site, TL_<50_ was independently associated with a higher risk of death among PF subjects. This pattern remained consistent across different racial/ethnic groups (Hispanic, HR = 3.40, 95% CI = 1.88–6.14; Asian, HR = 2.11, 95% CI = 0.54–8.23; White, HR = 2.21 95% CI = 1.79–2.72; and Black participants, HR = 2.22, 95% CI = 1.05–4.66).

## Discussion

Short LTLs are present in patients with PF and are associated with survival regardless of race/ethnicity. Distinct racial/ethnic differences exist in the extent of LTL shortening and in the correlation with chronologic age, supporting epidemiologic differences. Black subjects had a 3.6-fold increase in the odds of having longer LTL and were over a decade younger than White individuals at PF diagnosis. While LTL shortens with PF^9,14^, the results of this analysis show that this trend does not occur uniformly across all racial/ethnic groups but may reflect the chronological age at the time of PF diagnosis. To our knowledge, this original investigation uniquely adds to the literature in linking genomic markers to clinically identified epidemiological differences across patients with PF from diverse racial/ethnic groups.

The linear relationship between chronological age and telomere length is significantly disrupted in PF compared to healthy controls. The lack of congruence in this linear relationship amongst individuals with PF might suggest that acceleration of the biological aging processes, early senescence, and cellular apoptosis are contributory pathobiologic mechanisms underlying PF. We found that the shortest LTLs were present in older male Asian and White individuals with PF, and among these subjects, IPF was the most prevalent. Conversely, Black participants with PF had the least short LTL and the lowest prevalence of IPF. This supports the observation that telomere shortening may be causal for IPF^5^ and may explain why IPF is the most common form of PF encountered in kindred harboring pathogenic mutations in telomere-maintenance genes and short LTLs^15,16^. In contrast, we found that Black participants had less-short LTL and developed lung disease at a much younger chronological age, suggesting that pathologic alterations of telomere homeostasis may play a lesser role in PF development in this group^17^. These pathological alterations in telomere homeostasis, such as TERC or TERT mutations, may lead to defects in the telomerase complex^18^, and, subsequently, culminate in PF with very short telomeres. Comparatively, autoimmune and most other causes of PF do not typically result in such profoundly shortened telomeres. As telomere length is often not entirely dependent on genetic factors alone, but gene-by-environment factors, including smoking, environmental air pollution, and emotional stress, are often contributory, it is possible that when compared to White patients, LTL in Black patients are not as profoundly shortened at disease onset. It is also possible that the subset of black people in whom the LTL are not as profoundly shortened are more likely to have better medical follow-up (due to their social status, or from the presence of extrapulmonary symptoms) and therefore are more likely to have an earlier diagnosis of PF, and are for the same reasons at lower risk of death.

Notably, Black subjects had the highest prevalence of autoimmune-associated causes of PF, which was four times higher than that seen among White subjects and twice that of Asian and Hispanic subjects. This underscores the divergent contribution of lung stressors in the etiopathogenesis of PF across racial backgrounds. Non-hereditary factors such as inflammatory disease states, sporadic autoimmune conditions^19–21^, toxic inhalation of environmental antigens^22^, air pollution^23,24^, and various socioeconomic determinants of health^25^, are known to disproportionately impact Black individuals.

Black race has been associated with longer telomere length in healthy individuals across most tissues, including lungs and peripheral leukocytes^26,27^. However, the difference between Black and White subjects among those with PF in our study was several orders of magnitude higher than in controls reinforcing the idea that additional factors influence this disparity. Current formulas for age-based expected LTL appear to hold across diverse racial/ethnic groups and observed differences in LTL across races may reflect the cumulative effects of differential exposure to oxidative stress over the individual’s lifecourse^28,29^. Asians had the longest LTLs amongst healthy controls. However, in the pooled PF cohort, their LTL was almost as short as that of White subjects. This significant shortening of LTL in Asians with PF also suggests the influence of ethnic factors. Additionally, the high prevalence of IPF among Asians, which approaches seen in White populations, further supports this observation^30^.

In consolidating the plethora of evidence that points to LTL processes as being causal for PF, our results provide additional illumination by demonstrating that the magnitude of effect in this causal relationship may differ by subtype, as seen with autoimmune and idiopathic forms of PF in this study. The causal model of shorter LTL culminating in PF is further strengthened by our depiction of decreasing LTL as being directly linked to increased odds for PF, despite the variation in odds ratios observed across different racial groups.

Consistent with our study hypothesis, we observed a strong association of LTL with chronological age across racially and ethnically diverse PF cohorts. While many of the observed differences in LTL were related to age differences between subgroups, our study also demonstrated age-specific differences in the association of LTL with the risk of lung function impairment and specific PF subtypes across diverse races. As LTLs are not uniformly correlated with age across all age groups (Figs. 3, 4), it would be reasonable to expect that the association with clinical predictors of respiratory impairment would differ based on the age stratum. While our study evaluated LTLs in circulation, it has been shown that LTL measurements in peripheral blood correlate highly with that of the lungs^26^. White subjects in the seventh and eighth decades of life with shorter LTLs had an increased risk of lung function impairment. The observation that older Black or White individuals with PF and shorter LTLs were more likely to be male is consistent with data showing that telomerase’s in vitro activity is regulated by sex hormones, and circulating levels of sex hormones are associated with LTLs^31^. This is also consistent with population data showing that LTLs are shorter in males than females, highlighting the need for future studies to further examine the potential value of hormonal-based interventions in PF for ameliorating telomere loss or impacting clinically relevant outcomes^26,32^.

In recent studies demonstrating possible pharmacogenomic effects of short telomeres, we identified LTL as a biomarker that may identify subsets of patients with PF who are at risk for poor outcomes when exposed to immunosuppression^33,34^. The use of prednisone/azathioprine/N-acetylcysteine in patients who had IPF and LTL below the 10th percentile was associated with a higher composite endpoint of death, lung transplantation, hospitalization, or FVC decline^33^. In a separate study, we showed that mycophenolate therapy is not associated with improvement in survival or lung function among patients who have FHP and short telomeres^34^. However, in the absence of short LTL, mycophenolate therapy was associated with improved survival in FHP^34^. Given that the frequency and pretest probability of specific PF subtypes varies across populations, such as lower IPF prevalence among Black subjects and higher frequency of FHP among certain Asian subpopulations^35,36^, this knowledge of potential pharmacogenomic effects of short telomeres may prompt the measurement of LTL and influence the decision to treat as well as the choice of immunosuppressive therapy.

Despite LTL variation in this diverse PF population, we observed that shorter LTL is uniformly associated with worsened survival across all races independent of the PF subtype. As most genetic data stem from calculations derived primarily from a single race, their predictive accuracy, when applied to other racial/ethnic groups, has previously been uncertain. While the routine application of standardized reference LTL values that are derived primarily from white populations to all races may indeed result in decreased precision, our findings undermine the argument that inherent bias in genomic biomarkers entirely precludes their utility in other populations. Our findings of an overall negative relationship between mortality and LTL in non-White populations correspond to the already reported mortality in White populations as described in several previous investigations in the general population and appear to occur irrespective of the severity of fibrosis. We showed that among patients with PF, LTL measurements have substantial predictive value for mortality even after adjusting for known confounding variables. In our study, there was a “dose-dependent” relationship between shorter LTL in quartiles with mortality across racial/ethnic groups. Further, LTL below the median was a useful prognostic biomarker applicable to all racial/ethnic groups. Identifying at-risk individuals with short telomeres regardless of racial origin could inform pharmacotherapeutic interventions and guide targeted management, thus enhancing personalized medicine delivery to patients.

This study has some limitations. First, this cross-sectional cohort study was limited in its ability to assess LTL dynamics longitudinally. Second, our analyses were based on qPCR with measurements of the average LTL derived from the standard single-copy gene ratio. However, this technique allowed us to assess LTLs on archived samples. Third, our study is focused on differences across race/ethnic groups, complex social constructs that often reflect an individual’s perception of their familial origin, cultural environment, and genetic makeup^37^. Fourth, it is also possible that the observed association of short telomeres with a detrimental survival pattern is due to mortality from comorbidities in PF, including coronary artery disease, pulmonary hypertension, dyslipidemia, combined pulmonary fibrosis and emphysema, obstructive sleep apnea, gastro-esophageal reflux disease, among others^38–40^. Given that physiologic age (linked to short telomeres) may be more important than chronological age in driving mortality due to comorbidities, statistical adjustments for age would not necessarily account for these. However, given the retrospective nature of this analyses, we were unable to ascertain the cause of death for decedents in our study. Fifth, due to methodological heterogeneity in telomere length measurements, we could not directly quantify across-group averaged raw estimates of absolute LTLs between cases and controls, as data was measured differently between both populations and within different tissues^41,42^. However, using *z*-score standardization, we were able to show within-group racial/ethnic differences in standardized LTL and how these differences were compared across populations. Further, results among the Asian subgroup appeared less consistent. This study limitation likely stems from the consideration of all Asian participants as being in a single Asian ethnic group in the analyses despite substantial heterogeneity in factors such as socioeconomic position, cultural norms and behaviors, and immigration history across various Asian ethnicities. This limitation in data might have contributed to less precision in this subgroup as variations across ethnicities are averaged with their aggregate treatment. Additionally, our study utilized self-reported race for racial/ethnic categorizations and this precluded our ability to discuss potential genetic differences in risk of PF or PF-related mortality as we do not have genetic ancestry data with which to assess genetic differences. Finally, residual confounding variables that could influence LTLs to remain possible even after adjustment for identified factors. Nevertheless, the magnitude of our sample size and the widely disparate geographic regions covered should mitigate such potential effects.

Telomere length in subjects with PF is a heterogeneous but predictive biomarker that is associated with chronological age. Shorter LTL measurements hold promise for consistently identifying patients at the highest risk of death for earlier and more precise intervention irrespective of racial/ethnic background.

## Methods

### Study design, age, and mortality ascertainment

This cohort study used data obtained from patients prospectively enrolled with a confident multidisciplinary diagnosis of fibrotic interstitial lung disease subtypes, collectively referred to as PF, at four geographically disparate US tertiary centers: University of Chicago (UChicago), University of California San Francisco (UCSF), the University of California Davis (UC Davis), and University of Texas Southwestern, Dallas (UTSW) between September 2003 and December 2019. Within their respective centers, all study participants underwent a confident multidisciplinary evaluation of PF performed using available clinical data, pulmonary function tests, high-resolution computed tomography (HRCT) scans, and surgical lung biopsies according to current American Thoracic Society/European Respiratory Society criteria^43–47^. An assessment of the multidisciplinary diagnosis of ILD was performed by pulmonologists in conjunction with rheumatologists, dedicated thoracic radiologists, and a thoracic pathologist.

All participants were recruited using consensus American Thoracic Society (ATS)/European Respiratory Society (ERS) criteria^45,46^ and were enrolled after obtaining written informed consent. All relevant hospital institutional review boards approved the study (IRB:UChicago#14163 A;UCSF#10-01592 & #10-00198; UCDavis#585448-7 & #875917-2; UTSW#082010-127 & #AAAS0753). Study participants were enrolled at the diagnosis of PF. Participants enrolled in the Idiopathic Pulmonary Fibrosis Clinical Research Network (IPFnet)^48^ clinical trials(NCT00650091/NCT00957242) and consented to participate in the optional genetics substudy with available genomic DNA were included in this study. Healthy participants enrolled in the “Health and Retirement Study”(HRS)[*NIA-U01AG009740*]^49^ who had available individual-level demographic data and LTL measurements by quantitative polymerase chain reaction (qPCR) assay were included as controls.

Race and ethnicity data were collected by self-report in prespecified fixed categories, and chronological age was determined at study enrollment. Categorization of self-reported race was implemented per federally defined US Census Bureau standards on race (White, Black or African-American, American-Indian or Alaska Native, Asian and Native Hawaiian or Pacific Islander) and ethnicity (Hispanic or not Hispanic)^50^. Patients meeting these pre-defined White (not Hispanic), Black (not Hispanic), Asian (not Hispanic), or Hispanic racial/ethnic categories were included in the analysis. Race and ethnicity comparisons were made between Hispanic participants, non-Hispanic White participants, non-Hispanic Black participants, and non-Hispanic Asian participants (hereafter referred to as Hispanic, White, Black, and Asian participants, respectively). Vital status was determined by reviewing medical records and confirmed using the United States Social Security death index. Lung transplantation status was determined from the EMR of individual sites. Analyses of LTL association with mortality were adjusted for age and sex, as these are known to influence mortality in other pulmonary diseases. In all cohorts, mortality refers to all-cause mortality unless otherwise specified.

### Telomere length analysis

Genomic DNA was isolated from peripheral blood leukocytes obtained at study enrollment. Peripheral blood LTL was measured using qPCR and the RotoGene real-time PCR system (Qiagen) in triplicate^18,33,51,52^, and age-adjusted LTL was calculated using normal controls^34,51^. For comparative analyses, LTL was assessed in salivary leukocytes from HRS control subjects. We derived standardized LTL values from the observed (qPCR-based) LTL minus the Cronkhite 2008 expected value and depicted these in the results as telomere length (adjusted O–E)^51,52^. LTL measurements are depicted as a T/S ratio for all cohorts except for California—where LTL in base pairs (bp) is reported. Given the potential variance in qPCR-based LTL measurement across study sites, standardized LTL values were calculated by applying z-score normalization with multivariable adjustment for age and sex, and categorization into quartiles^53^. This conversion enabled each sample to have the same distribution based on standard deviations empirically computed for each individual across each cohort^54^. In survival analyses, we used LTL below the median (TL50) and transformed TL (negative log-transformed inverse of one minus percentile TL), comparing mortality hazard ratios for each centile of TL to the highest TL centile among subjects with PF.

### Statistical analyses

In all cohorts, association analyses between pairs of variables were conducted using Fisher exact tests for categorical variables and two-tailed *t*-tests or analysis of variance (ANOVA) for continuous variables. Comparisons of LTL between independent diagnostic subgroups of PF were conducted using the nonparametric Mood’s median test. In sensitivity analyses to adjust for baseline demographic differences between Black and White participants with PF, propensity-score matching was performed in this selected subcohort and LTL measurements analyzed. Generalized linear models with a logistic regression link were used to evaluate the age-LTL relationship. The linearity assumption was assessed for continuous covariates using a lack-of-fit sum of squares test that compared the linear fit to restricted cubic spline fit with five knots, which ascertains that statistical models fit well with the selected set of observations. Linear regression models were fitted to assess congruence between median standardized LTL and chronological age for all included racial/ethnic groups stratified by sex, while rates of absolute mortality were analyzed using conditional logistic regression. Odds ratios for LTL comparisons across racial/ethnic groups were generated from transformed coefficients of linear combinations.

Cox proportional hazards models were used for hazard ratio estimation and analysis of time to mortality with robust standard errors to account for familial correlation within the cohort. All included patients were stratified by quartiles of standardized LTLs and the Cochran–Armitage test was used to assess trends across quartiles, using quartile integers (1, 2, 3, and 4) as scores^18,54^. The estimated survival functions for each quartile were plotted based on the Cox model. For the PF cohort, we assessed transplant-free survival over five years, and transplant-free survival time was calculated from study enrollment to death, lung transplantation, loss to follow-up, or end of the study period. Survival time was censored on December 31, 2019, or when a participant was lost to follow-up. In multivariable models assessing mortality, we sought to only adjust for established confounders previously linked to both the predictor and the outcome; therefore, covariate selection for inclusion in the model was based on potential confounding variables known to affect telomere length and PF mortality^55^. As smoking and BMI are inconsistently associated with mortality in interstitial lung disease^56–58^, and when present, seem to exert their effect on worsened PF mortality through worsening pulmonary function or accelerating the progression of fibrosis^59,60^, the analyses of all multivariable outcome models were adjusted for age, sex, measures of pulmonary function including forced vital capacity (FVC) and diffusing capacity of the lungs for carbon monoxide (DL_CO_), and hospital center. The relationship between LTL and the severity of PF using the most widely-used index for assessing PF severity, the ILD-GAP score^61^. Survival curves are plotted using the Kaplan–Meier survival estimator. In Cox models, we assessed all covariate effects over time and none violated the proportional hazards assumption.

All *P* values were two-sided, and a level of 0.05 was considered statistically significant. Data collation was performed using Microsoft Excel for Mac Version 16.65, 2019. Analyses were conducted using Stata (V 2019.R.16, V 2021.R17; StataCorp) and R.v.3.5.1.

### Reporting summary

All sexes were considered in this observational cohort study and the sex of participants was determined based on self-report as documented within the respective data registries. No consent was obtained for reporting and sharing individual-level data. Results of sex-based analyses, which were performed a-priori are reported accordingly. Further information on research design is available in the Nature Portfolio Reporting Summary linked to this article.

## Supplementary information

Supplementary Information

Reporting Summary

## Data Availability

All data supporting the findings described in this manuscript are available in the article and in the Supplementary Information and from the corresponding author upon request. Source data are provided with this paper. The processed telomere length data for the HRS control population are available at “hrsdata” with the identifier [https://hrsdata.isr.umich.edu/data-products/2008-telomere-data]. Response to data requests will be made within 6–8 weeks of receipt. The pulmonary fibrosis telomere length data that support the findings of this study are subject to controlled access via data use agreements due to reasons of clinical human data sensitivity and are available upon reasonable request from the corresponding author [A.A.].

## Code availability

Code for data cleaning and analysis is provided as part of the replication package^62^. It is available at 10.5281/zenodo.7431322.
