# Supplementary material for: Telomere length associates with chronological age and mortality across racially diverse pulmonary fibrosis cohorts
Source: Nat Commun. 2023 Mar 17;14:1489. doi: 10.1038/s41467-023-37193-6 (PMC10023792; doi:10.1038/s41467-023-37193-6)
Supplement: Supplementary file 1 — Supplementary Information [file 41467_2023_37193_MOESM1_ESM.pdf]

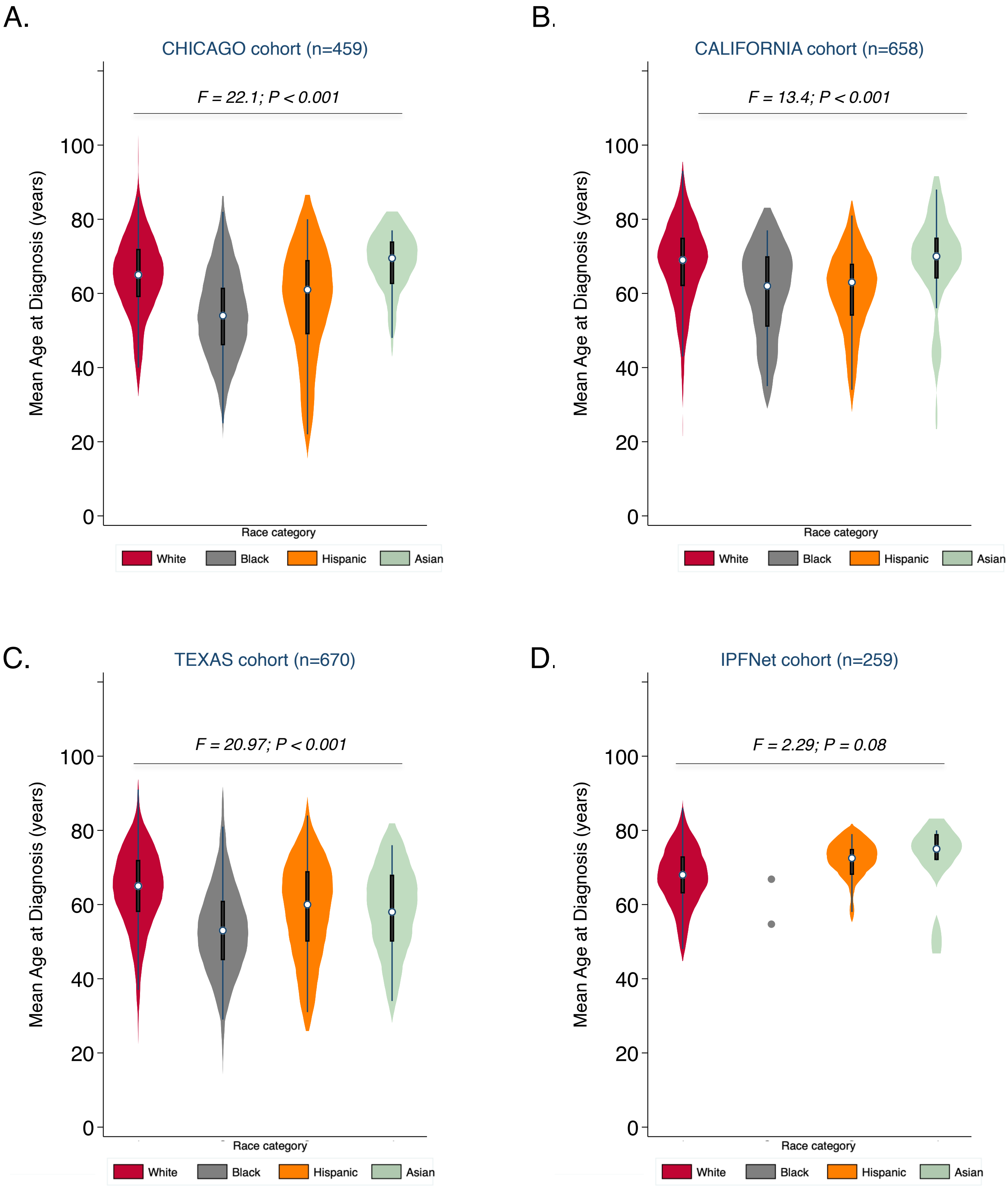

Supplementary Figure 1. Mean age at diagnosis of pulmonary fibrosis is lowest among Black subjects. Stratification by racial subgroup depicts **(A)** The CHICAGO cohort (n=459, White *red*, n=332; Black *grey*, n=84; Hispanic *orange*, n=35; and Asian *green*, n=8); **(B)** The CALIFORNIA cohort (n=646, White *red*, n=517; Black *grey*, n=21; Hispanic *orange*, n=70; and Asian *green*, n=36); **(C)** The TEXAS cohort (n=670, White *red*, n=528; Black *grey*, n=55; Hispanic *orange*, n=68; and Asian *green*, n=19); and **(D)** The *IPFNet* cohort (n=259, White *red*, n=236; Black *grey*, n=2; Hispanic *orange*, n=14; and Asian *green*, n=7). Group comparisons across racial subgroups conducted using Bartlett's one-way analysis of variance (ANOVA) test. In the violin plots, the white circle represents the median of the data; the black vertical box indicates the interquartile range; the thin blue vertical line indicates 1.5x the interquartile range and the density curves depicts the distribution of numeric data for all sample points within each group.

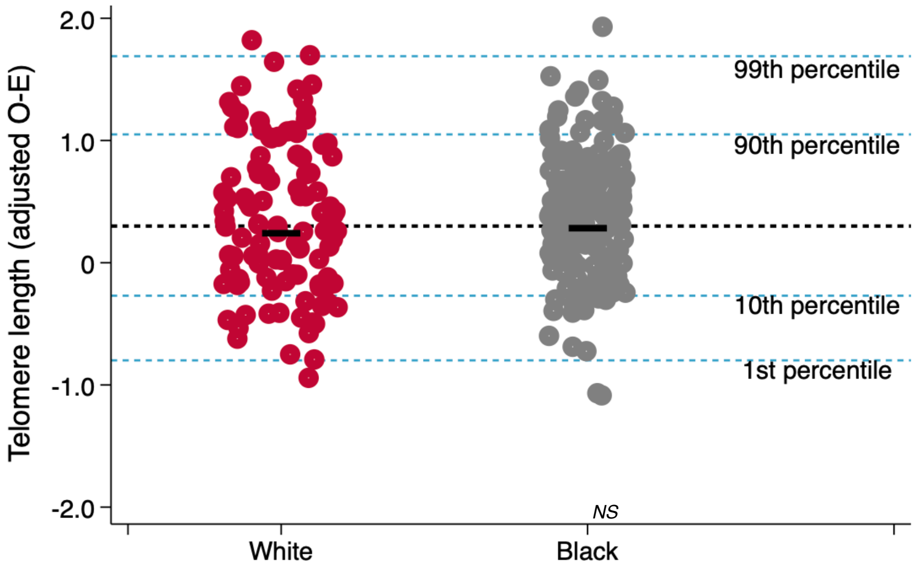

Supplementary Figure 2. Mean observed minus expected (O–E; age and gender-adjusted) leukocyte telomere length (TL) is similar in White subjects propensity-matched to Black subjects with pulmonary fibrosis (PF). Mean age and gender-adjusted TL (O–E) of 0·33 (0·56) in White subjects (n=106), and 0·37 (0·49) in Black subjects (n=162), respectively. Thick short black lines show the median for each subgroup. The black dotted line shows the median TL; blue dotted lines show approximate age-adjusted prediction bands in percentiles for each cohort. *Group comparisons between white subjects (#) and other racial subgroups conducted using the two-sided student's T-test;* NS=not significant ($P \geq 0{\cdot}05$).

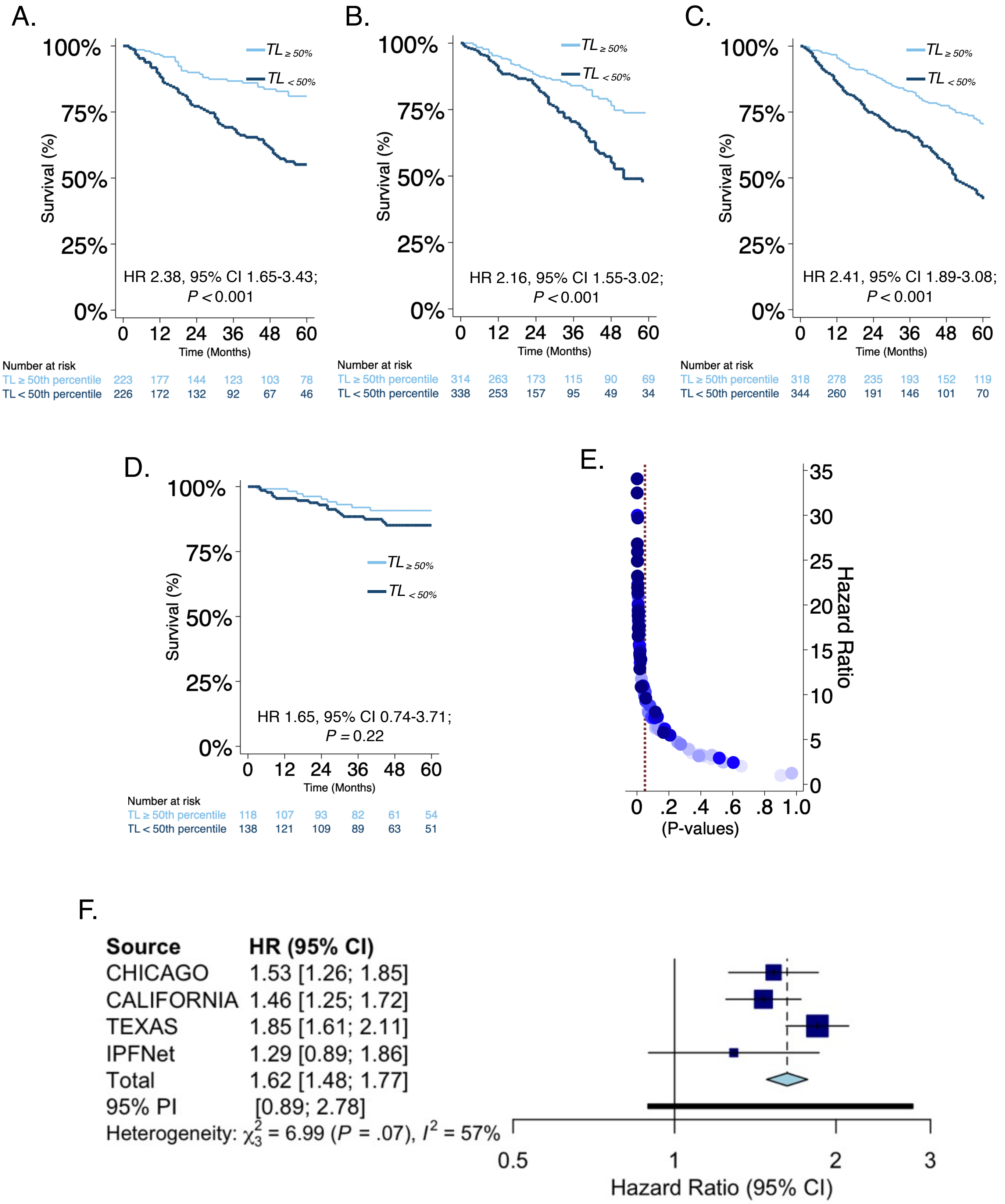

Supplementary Figure 3. Shorter leukocyte telomere length (TL) consistently predicts worse survival patterns in pulmonary fibrosis. Survival stratified by age and gender-adjusted TL below the median ($TL_{<50\%}$) vs. above the median ($TL_{\geq 50\%}$) in **(A)** University of Chicago (CHICAGO) cohort ; **(B)** University of California, Davis and University of California, San Francisco (CALIFORNIA) cohort; **(C)** University of Texas, Southwestern (UTSW) cohort; **(D)** Idiopathic Pulmonary Fibrosis Network Clinical Trials (IPFNet) cohort. Unadjusted Cox proportional hazard ratio (HR) and 95% confidence interval of this mortality hazard estimate depicted with its respective P-value for each cohort. **(E)** Plot of p-values for the association of mortality hazard ratios (HR) in PF with transformed TL (negative log-transformed inverse of one minus percentile TL); HR depicted for decreasing centiles of TL compared to highest centile of TL. **(F)** Survival stratified by age and gender-adjusted TL in quartiles for each center; CHICAGO (n=459), CALIFORNIA (n=658), TEXAS (n=670), IPFNET (n=259); TOTAL (n=2046). *The navy blue boxes within the forest plot represents the point estimate for the mortality hazard ratio for each cohort, the thin horizontal line represents its 95% confidence interval, the vertical line is the line of no effect, and the diamond represents the overall effect estimate.*

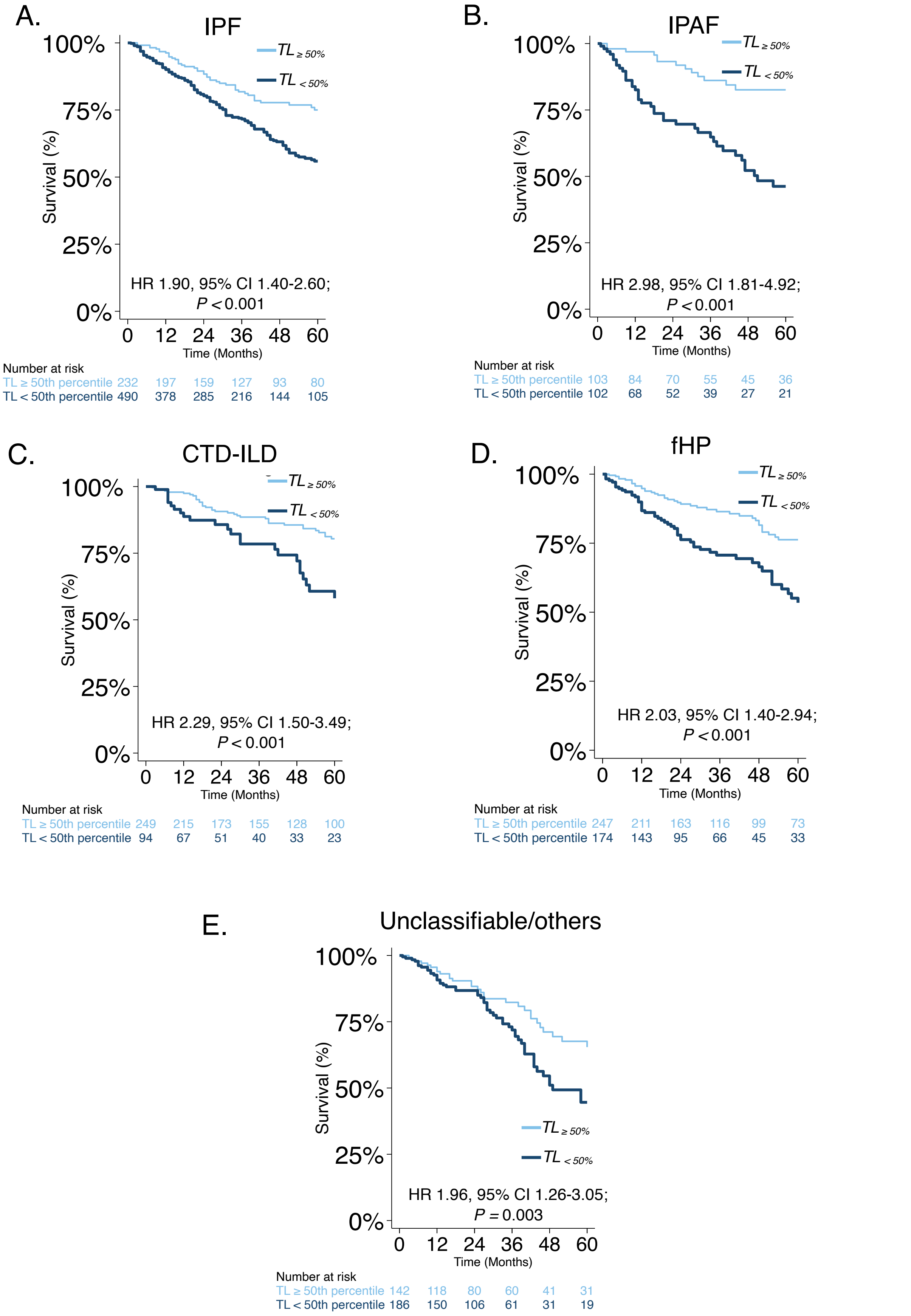

Supplementary Figure 4. Shorter leukocyte telomere length (TL) consistently predicts worse survival patterns in pulmonary fibrosis (PF) subtypes. Survival stratified by age and gender-adjusted TL below the median ($TL_{<50\%}$) vs. above the median ($TL_{\geq 50\%}$) in **(A) i**diopathic pulmonary fibrosis (IPF); **(B)** interstitial pneumonia with autoimmune features (IPAF); **(C)** connective tissue disease-related interstitial lung disease (CTD-ILD); **(D)** fibrotic hypersensitivity pneumonitis (fHP) **(E)** unclassifiable or other PF subtypes. Unadjusted Cox proportional hazard ratio (HR) and 95% confidence interval of this mortality hazard estimate depicted with its respective P-value for each PF subtype.

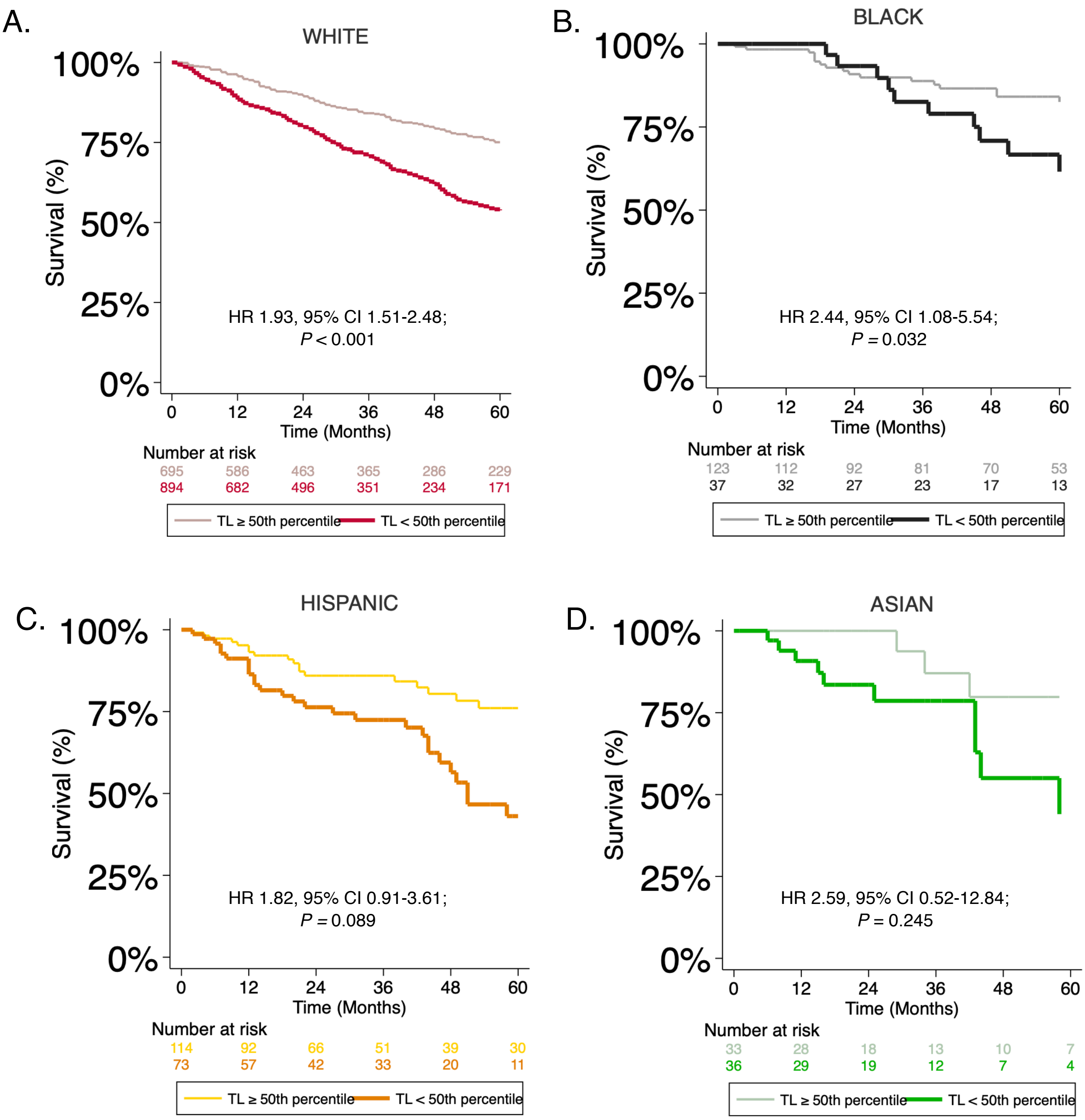

Supplementary Figure 5. Leukocyte Telomere Length (TL) below the 50th percentile consistently predicts worse survival across diverse racial groups with pulmonary fibrosis. Survival pattern by *TL* in **(A)** White subjects; **(B)** Black subjects; **(C)** Hispanic subjects; and **(D)** Asian subjects. Unadjusted Cox proportional hazard ratio (HR) and 95% confidence interval of this mortality hazard estimate depicted with its respective P-value for each racial/ethnic group.

Supplementary Table 1. Characteristics of Study Participants with Pulmonary Fibrosis

**Characteristics***	**CHICAGO (n=459)**	**CALIFORNIA (n=658)**	**TEXAS (n=670)**	**IPFNET (n=259)**	***P*-value#**
Age, years	62.5 (11.8)	66.9 (11.3)	62.4 (11.6)	67.7 (7.8)	< 0.001
Male	225 (49.0)	318 (48.3)	364 (54.3)	191 (73.8)	< 0.001
Race/Ethnicity					
White	332 (72.3)	517 (78.6)	528 (78.8)	236 (91.1)	< 0.001
Black	84 (18.3)	21 (3.2)	55 (8.2)	2 (0.8)	< 0.001
Hispanic	35 (7.6)	70 (10.6)	68 (10.2)	14 (5.4)	0.042
Asian	8 (1.7)	36 (5.5)	19 (2.8)	7 (2.7)	0.004
Ever Smoker	273 (60.0)	362 (55.1)	353 (52.7)	189 (73.0)	< 0.001
Lung Function					
FVC (% predicted)	64.2 (18.5)	69.6 (19.6)	66.5 (19.9)	66.3 (16.9)	< 0.001
DL_{CO} (% predicted)	51.6 (20.9)	49.5 (18.7)	45.3 (18.9)	40.7 (13.0)	< 0.001
ILD sub-category					
IPF	120 (26.1)	68 (10.3)	288 (43.0)	259 (100)	< 0.001
IPAF	115 (25.1)	21 (3.2)	71 (10.6)	0 (0)	< 0.001
CTD-ILD	146 (31.8)	48 (7.3)	155 (23.1)	0 (0)	< 0.001
FHP	77 (16.8)	261 (39.7)	84 (12.5)	0 (0)	< 0.001
Unclassifiable/Others	1 (0.2)	260 (39.5)	72 (10.8)	0 (0)	<0.001
Mean survival time, mths (95% CI)	102 (94 -110)	99 (91-107)	101 (90-113)	72 (70-75)	< 0.001
Number of deaths, n (%)	129 (28.4)	154 (23.4)	288 (43.0)	26 (10.0)	< 0.001
Lung transplantation, n (%)	32 (7.0)	17 (2.6)	125 (18.7)	5 (1.9)	< 0.001

*Total sample size, n=2,042. *Categorical variables presented as n (%); continuous variables presented as means (SD). #P-value* for chi-squared (categorical data) or one-way ANOVA (continuous data) *comparing the CHICAGO, CALIFORNIA, TEXAS, and IPFNET cohorts. Exception for participants; smoking status, n=2041; FVC=forced vital capacity, n=2015;* DL_{CO}*=diffusing capacity of the lungs, n=1959; ILD=interstitial lung disease; IPF=Idiopathic pulmonary fibrosis, n=735; IPAF=interstitial pneumonia with autoimmune features, n=207; CTD-ILD=Connective tissue disease associated-ILD, n=349; FHP=Fibrotic hypersensitivity pneumonitis, n=422; Unclassifiable/Others, n=333.*

Supplementary Table 2. Mean Age and Telomere Lengths Differences Between White and Black Subjects

Characteristic	WHITE (n=1613)	BLACK (n=162)	Absolute Mean Difference (Δ)	Delta ratio (95% CI)	*P*-value#
*Mean TL**					
PF cohort	-0·06 (0·47)	0·37 (0·49)	0.43 (0.48)	3.58 (2.56 – 5.37)	< 0.001
HRS cohort	-0·04 (0·49)	0·08 (0·48)	0.12 (0.48)		
Age, years					
PF cohort	66.02 (10.40)	54.93 (12.20)	11.09 (36.74)	4.70 (3.37 – 7.17)	< 0.001
HRS cohort	70.19 (10.23)	67.83 (9.90)	2.36 (28.27)		

**Standardized mean observed minus expected (O–E) leukocyte telomere length (TL) across study population presented as means (SD).*

#P-value for the student's T-test comparing Δ between PF and HRS cohorts. PF=pulmonary fibrosis; HRS=Health Retirement Survey.

Supplementary Table 3. Comparison of Mean Leukocyte Telomere Lengths Depicting Similarities Across Study Participants with Pulmonary Fibrosis Stratified by Race

Characteristics*	CHICAGO (n=459)	CALIFORNIA (n=658)	TEXAS (n=670)	IPFNET (n=259)	*P*-value#
White					
Q1 *(n=435)*	-0.64 (0.29)	-0.66 (0.32)	-0.62 (0.27)	-0.61 (0.32)	0.660
Q2 *(n=426)*	-0.14 (0.10)	-0.14 (0.09)	-0.15 (0.10)	-0.13 (0.09)	0.456
Q3 *(n=386)*	0.16 (0.09)	0.17 (0.09)	0.16 (0.08)	0.13 (0.07)	0.012
Q4 *(n=345)*	0.55 (0.23)	0.59 (0.22)	0.60 (0.25)	0.61 (0.31)	0.590
Black					
Q1 *(n=16)*	-0.60 (0.15)	-0.44 (0.01)	-0.46 (0.05)	---	0.117
Q2 *(n=25)*	-0.11 (0.12)	-0.15 (0.14)	-0.13 (0.06)	-0.17 (0.00)	0.875
Q3 *(n=44)*	0.16 (0.07)	0.17 (0.07)	0.17 (0.09)	---	0.954
Q4 *(n=77)*	0.65 (0.24)	0.69 (0.30)	0.64 (0.27)	1.28 (0.00)	0.127
Hispanic					
Q1 *(n=38)*	-0.77 (0.43)	-0.57 (0.25)	-0.64 (0.38)	-0.71 (0.16)	0.557
Q2 *(n=38)*	-0.09 (0.09)	-0.11 (0.09)	-0.13 (0.10)	-0.15 (0.11)	0.786
Q3 *(n=50)*	0.21 (0.07)	0.18 (0.11)	0.18 (0.09)	0.23 (0.04)	0.704
Q4 *(n=61)*	0.76 (0.29)	0.66 (0.24)	0.64 (0.30)	0.63 (0.15)	0.601
Asian					
Q1 *(n=13)*	-0.64 (0.00)	-0.60 (0.31)	-0.81 (0.09)	-0.50 (0.00)	0.577
Q2 *(n=14)*	---	-0.13 (0.11)	-0.14 (0.11)	-0.27 (0.00)	0.511
Q3 *(n=21)*	0.18 (0.07)	0.15 (0.08)	0.17 (0.10)	0.16 (0.00)	0.922
Q4 *(n=22)*	0.52 (0.18)	0.74 (0.32)	0.73 (0.16)	0.45 (0.21)	0.246

*Total sample size, n=1,065. *Standardized telomere length across study population presented as means (SD) and categorized by quartiles, Q1=1st quartile, Q2=2nd quartile, Q3=3rd quartile, Q4=4th quartile. Telomere lengths unadjusted for age or sex.*

#P-value for ANOVA comparisons between the CHICAGO, CALIFORNIA, TEXAS, and IPFNET cohorts. White n=784, Black n=102; Hispanic n=104; Asian n=42; Other race (not depicted) n=14.

Supplementary Table 4. Differences in Telomere Lengths Stratified by Race and Sex*

Characteristics	WHITE	BLACK	HISPANIC	ASIAN	*P*-value#
**CHICAGO**	**(n=332)**	**(n=84)**	**(n=35)**	**(n=8)**	
All subjects	-0.09 (0.47)	0.29 (0.45)	0.14 (0.62)	0.16 (0.37)	<0.001
Male	-0.14 (0.46)	0.13 (0.53)	0.07 (0.34)	0.03 (0.46)	0.032
Female	-0.03 (0.47)	0.35 (0.41)	0.18 (0.73)	0.30 (0.24)	<0.001
**CALIFORNIA**	**(n=517)**	**(n=21)**	**(n=70)**	**(n=36)**	
All subjects	-0.03 (0.50)	0.20 (0.40)	0.07 (0.50)	0.10 (0.53)	0.115
Male	-0.07 (0.49)	0.03 (0.36)	0.04 (0.57)	-0.14 (0.50)	0.748
Female	0.02 (0.51)	0.29 (0.41)	0.08 (0.45)	0.40 (0.40)	0.015
**TEXAS**	**(n=528)**	**(n=55)**	**(n=68)**	**(n=19)**	
All subjects	-0.05 (0.49)	0.30 (0.45)	0.15 (0.50)	0.08 (0.59)	<0.001
Male	-0.08 (0.47)	0.07 (0.37)	0.05 (0.49)	0.01 (0.56)	0.269
Female	-0.02 (0.51)	0.37 (0.45)	0.32 (0.49)	0.15 (0.64)	<0.001
**IPFNET**	**(n=236)**	**(n=2)**	**(n=14)**	**(n=7)**	
All subjects	-0.02 (0.49)	0.55 (1.02)	0.12 (0.56)	0.17 (0.43)	0.228
Male	-0.03 (0.43)	0.55 (1.02)	0.11 (0.54)	0.07 (0.44)	0.246
Female	0.01 (0.64)	---	0.14 (0.63)	0.43 (0.38)	0.616
**COMBINED**	**(n=1613)**	**(n=162)**	**(n=187)**	**(n=70)**	
All subjects	-0.05 (0.49)	0.29 (0.45)	0.11 (0.52)	0.11 (0.51)	<0.001
Male	-0.08 (0.47)	0.12 (0.48)	0.05 (0.50)	-0.06 (0.49)	0.012
Female	0.00 (0.51)	0.35 (0.42)	0.17 (0.55)	0.31 (0.46)	<0.001
**HRS COHORT**	**(n=4319)**	**(n=779)**	**(n=614)**	**(n=96)**	
All subjects	-0.03 (0.45)	0.13 (0.76)	0.02 (0.41)	0.01 (0.43)	<0.001
Male	-0.02 (0.53)	0.11 (0.59)	0.02 (0.56)	-0.03 (0.27)	0.001
Female	-0.03 (0.37)	0.14 (0.84)	0.02 (0.28)	0.03 (0.51)	<0.001

*Total sample size, n=7,854. *Standardized telomere length across study population presented as means (SD). Telomere lengths unadjusted for age or sex. #P-value for ANOVA test comparing all four main racial groups. Patients with mixed or other racial ancestry not depicted above, n=14.*

Supplementary Table 5. Demographics of Pulmonary Fibrosis (PF) cases stratified by subtype

**Characteristics***	**IPF**	**IPAF**	**CTD-ILD**	**FHP**	**Unclassifiable /Others**	***P*-value#**
PF (n=2046)	**(n=735)**	**(n=207)**	**(n=349)**	**(n=422)**	**(n=333)**	
Age, years	68.2 (8.5)	62.3 (11.1)	56.4 (13.1)	64.4 (10.4)	66.6 (11.8)	<0.001
Male	543 (73.9)	97 (46.9)	97 (27.8)	184 (43.6)	177 (53.2)	<0.001
Ever Smoker	508 (69.5)	113 (54.6)	145 (41.6)	207 (49.1)	204 (61.5)	<0.001
Body Mass Index	29.0 (5.2)	30.4 (7.0)	28.4 (6.5)	29.5 (6.1)	29.4 (5.9)	0.003
Lung Function						
FVC (% predicted)	67.3 (18.4)	63.9 (19.0)	66.6 (19.5)	66.1 (18.9)	69.7 (21.1)	0.012
FEV_1 (% predicted)	75.0 (19.7)	71.3 (20.4)	70.2 (20.6)	68.9 (19.9)	71.0 (21.3)	0.003
DL_{CO} (% predicted)	45.0 (17.6)	47.0 (18.0)	49.5 (20.6)	49.6 (19.2)	48.4 (19.8)	<0.001

*Categorical variables presented as n (%); continuous variables presented as means (SD). #P-value for chi-squared (categorical data) or one-way ANOVA (continuous data) comparing all four major PF subtypes. Exception for participants with: smoking status, n=2041; Body mass index=1505; FVC=forced vital capacity, n=2015; FEV1=forced expiratory volume in 1st second, n=1254; DL_{CO}=diffusing capacity of the lungs, n=1959. ILD=interstitial lung disease; IPF=idiopathic pulmonary fibrosis, n=735; IPAF=interstitial pneumonia with autoimmune features, n=207; CTD-ILD=connective tissue disease associated-ILD, n=349; fHP=fibrotic hypersensitivity pneumonitis, n=422; unclassifiable/other ILD, n=333.

Supplementary Table 6. Differences in Telomere Lengths Stratified by Race and PF Subtype*

Characteristics	WHITE	BLACK	HISPANIC	ASIAN	*P*-value#
IPF	**(n=653)**<br>-0.11 (0.49)	**(n=13)**<br>0.21 (0.52)	**(n=49)**<br>0.05 (0.47)	**(n=20)**<br>-0.06 (0.53)	0.018
IPAF	**(n=153)**<br>-0.04 (0.46)	**(n=30)**<br>0.29 (0.39)	**(n=15)**<br>-0.02 (0.71)	**(n=8)**<br>0.25 (0.29)	0.003
CTD-ILD	**(n=200)**<br>0.12 (0.46)	**(n=89)**<br>0.36 (0.38)	**(n=46)**<br>0.32 (0.45)	**(n=14)**<br>0.43 (0.38)	<0.001
FHP	**(n=359)**<br>-0.04 (0.51)	**(n=14)**<br>0.05 (0.65)	**(n=34)**<br>0.13 (0.46)	**(n=11)**<br>0.04 (0.64)	0.41
Unclassifiable/Others	**(n=248)**<br>-0.03 (0.49)	**(n=16)**<br>0.17 (0.56)	**(n=43)**<br>-0.00 (0.58)	**(n=17)**<br>0.02 (0.48)	0.65

*Total sample size, n=7,854. *Standardized telomere length across study population presented as means (SD). Telomere lengths unadjusted for age or sex. #P-value for ANOVA test comparing all four main racial groups. Patients with mixed or other racial ancestry not depicted above, n=14. PF=Pulmonary fibrosis; ILD=interstitial lung disease; IPF=Idiopathic pulmonary fibrosis, n=735; IPAF=interstitial pneumonia with autoimmune features, n=206; CTD-ILD=Connective tissue disease associated-ILD, n=349; FHP=Fibrotic hypersensitivity pneumonitis, n=418; Unclassifiable/Others, n=324.*

Supplementary Table 7. Generalized Model Assessing Age/Telomere Length Relationship Between PF and HRS Control Subjects Across Racial/Ethnic Categories

**Cohort**	**Beta coefficient**	***z***	**95% CI**	***P*-value**	***P*-interaction***
**WHITE**					
Age, years	-0·048	-276.36	-0.0485 to -0.0479	< 0.001	< 0.001
PF	-0·740	-32.91	-0.7844 to -0.6963	< 0.001	
**BLACK**					
Age, years	-0·048	-187.33	-0.0487 to -0.0477	< 0.001	< 0.001
PF	-0·885	-28.31	-0.9462 to -0.8236	< 0.001	
**HISPANIC**					
Age, years	-0·048	-118.17	-0.0490 to -0.0474	< 0.001	< 0.001
PF	-0·842	-18.30	-0.9317 to -0.7514	< 0.001	
**ASIAN**					
Age, years	-0·048	-36.68	-0.0509 to -0.0457	< 0.001	< 0.001
PF	-0·678	-5.48	-0.9206 to -0.4353	< 0.001	

PF=pulmonary fibrosis; HRS=Health Retirement Survey. White n=5,932, Black n=941, Hispanic n=801, Asian n=166, others n=14, All patients n=7854. Statistical test: generalized linear regression model assessing the age/telomere length relationship across racial/ethnic groups with P-value, beta coefficient, z-score, 95% confidence interval, and P-value for interaction (P-interaction) between PF and controls in the age/LTL relationship assessed and reported for each racial/ethnic group.

Supplementary Table 8. Stratification by Age Group Demonstrates Shorter Mean and Median Leukocyte Telomere Length with Increasing Chronological Age among Pulmonary Fibrosis Cohorts and Control Subjects.

**Characteristics***	Mean Telomere Length (SD)	Median Telomere Length (IQR)
**CHICAGO (n=459)**		
Less than 40yrs *(n=19)*	0.59 (0.40)	0.66 (0.63)
40 – 49yrs *(n=46)*	0.30 (0.40)	0.20 (0.50)
50 – 59yrs *(n=101)*	0.11 (0.47)	0.14 (0.44)
60 – 69yrs *(n=158)*	-0.06 (0.48)	-0.02 (0.63)
70yrs or older *(n=135)*	-0.20 (0.46)	-0.22 (0.54)
**CALIFORNIA (n=658)**		
Less than 40yrs *(n=12)*	0.52 (0.34)	0.45 (0.66)
40 – 49yrs *(n=45)*	0.19 (0.57)	0.17 (0.53)
50 – 59yrs *(n=91)*	0.04 (0.50)	0.12 (0.57)
60 – 69yrs *(n=215)*	0.01 (0.47)	0.06 (0.60)
70yrs or older *(n=295)*	-0.07 (0.50)	-0.10 (0.65)
**TEXAS (n=670)**		
Less than 40yrs *(n=31)*	0.37 (0.58)	0.42 (0.69)
40 – 49yrs *(n=61)*	0.32 (0.45)	0.39 (0.49)
50 – 59yrs *(n=148)*	0.06 (0.49)	0.11 (0.62)
60 – 69yrs *(n=240)*	-0.05 (0.49)	-0.03 (0.59)
70yrs or older *(n=190)*	-0.15 (0.45)	-0.17 (0.61)
**IPFNET (n=259)**		
Less than 40yrs *(n=0)*	---	---
40 – 49yrs *(n=3)*	0.52 (0.72)	0.88 (1.30)
50 – 59yrs *(n=37)*	-0.05 (0.67)	-0.00 (0.68)
60 – 69yrs *(n=114)*	-0.01 (0.50)	0.03 (0.51)
70yrs or older *(n=105)*	0.02 (0.42)	0.02 (0.55)
**HRS (n=5808)**		
Less than 40yrs *(n=14)*	0.02 (0.19)	-0.01 (0.21)
40 – 49yrs *(n=93)*	0.05 (0.27)	0.02 (0.25)
50 – 59yrs *(n=1056)*	0.04 (0.43)	-0.02 (0.26)
60 – 69yrs *(n=1766)*	0.01 (0.46)	-0.06 (0.26)
70yrs or older *(n=2879)*	-0.02 (0.55)	-0.09 (0.25)

*Total sample size, n=7,854. *Standardized telomere length across study population presented as means (SD). Telomere lengths unadjusted for age or sex.*

Supplementary Table 9. Models depicting association of leukocyte telomere length (LTL) with age stratified by sex across diverse racial populations.

Characteristics	WHITE	BLACK	HISPANIC	ASIAN	COMBINED
**Regression Models for Age***					
Unadjusted LTL					
PF Cohort	*(n=1613)*	*(n=162)*	*(n=187)*	*(n=70)*	*(n=2046)*
Male, *R* (*Root MSE*)	-0.18 (0.26)	-0.30 (0.22)	-0.37 (0.25)	-0.24 (0.29)	-0.22 (0.26)
95% CI	-0.25 to -0.12	-0.55 to 0.01⁺	-0.54 to -0.18	-0.53 to 0.09⁺	-0.28 to -0.16
Female, *R* (*Root MSE*)	-0.22 (0.29)	-0.28 (0.24)	-0.37 (0.30)	-0.42 (0.27)	-0.32 (0.29)
95% CI	-0.29 to -0.15	-0.44 to -0.10	-0.53 to -0.18	-0.69 to -0.06	-0.37 to -0.26
All, *R* (*Root MSE*)	-0.21 (0.27)	-0.29 (0.24)	-0.39 (0.28)	-0.17 (0.29)	-0.30 (0.28)
95% CI	-0.26 to -0.17	-0.43 to -0.14	-0.51 to -0.26	-0.40 to 0.07⁺	-0.33 to -0.26
HRS Cohort	*(n=4319)*	*(n=779)*	*(n=614)*	*(n=96)*	*(n=5808)*
Male, *R* (*Root MSE*)	-0.03 (0.78)	0.03 (0.86)	-0.12 (0.81)	-0.29 (0.38)	-0.04 (0.79)
95% CI	-0.08 to 0.01⁺	-0.09 to 0.15⁺	-0.23 to -0.02	-0.56 to 0.02⁺	-0.08 to -0.00
Female, *R* (*Root MSE*)	-0.05 (0.55)	-0.04 (1.23)	-0.16 (0.40)	-0.01 (0.76)	-0.06 (0.68)
95% CI	-0.10 to -0.01	-0.13 to 0.05⁺	-0.26 to -0.06	-0.25 to 0.27⁺	-0.10 to -0.03
All, *R* (*Root MSE*)	-0.04 (0.65)	-0.02 (1.11)	-0.12 (0.59)	-0.07 (0.63)	-0.05 (0.73)
95% CI	-0.07 to -0.01	-0.10 to -0.05	-0.42 to -0.05	-0.26 to -0.14	-0.08 to -0.03
Standardized LTL*					
PF Cohort	*(n=1613)*	*(n=162)*	*(n=187)*	*(n=70)*	*(n=2046)*
Male, *R* (*Root MSE*)	-0.17 (1.08)	-0.37 (1.08)	-0.35 (1.06)	-0.17 (1.09)	-0.21 (1.08)
95% CI	-0.24 to -0.11	-0.60 to -0.08	-0.52 to -0.16	-0.15 to 0.47⁺	-0.26 to -0.15
Female, *R* (*Root MSE*)	-0.26 (1.07)	-0.30 (0.88)	-0.26 (1.09)	-0.26 (0.95)	-0.31 (1.06)
95% CI	-0.32 to -0.19	-0.45 to -0.12	-0.44 to -0.06	-0.57 to 0.10⁺	-0.37 to -0.25
All, *R* (*Root MSE*)	-0.22 (1.08)	-0.33 (0.96)	-0.31 (1.07)	-0.14 (1.10)	-0.28 (1.08)
95% CI	-0.27 to -0.17	-0.46 to -0.18	-0.44 to -0.18	-0.36 to 0.10⁺	-0.32 to -0.24
HRS Cohort	*(n=4319)*	*(n=779)*	*(n=614)*	*(n=96)*	*(n=5808)*
Male, *R* (*Root MSE*)	-0.12 (1.10)	-0.09 (1.13)	-0.31 (1.13)	-0.39 (1.06)	-0.15 (1.11)
95% CI	-0.16 to -0.07	-0.21 to 0.02⁺	-0.42 to -0.19	-0.63 to -0.09	-0.19 to -0.11
Female, *R* (*Root MSE*)	-0.14 (1.08)	-0.18 (1.08)	-0.26 (1.06)	-0.31 (1.12)	-0.18 (1.09)
95% CI	-0.18 to -0.10	-0.27 to -0.10	-0.36 to -0.17	-0.53 to -0.06	-0.21 to -0.14
All, *R* (*Root MSE*)	-0.13 (1.09)	-0.15 (1.10)	-0.29 (1.09)	-0.35 (1.09)	-0.17 (1.10)
95% CI	-0.16 to -0.10	-0.22 to -0.09	-0.36 to -0.21	-0.51 to -0.16	-0.19 to -0.14

R= Pearson's bivariate correlation coefficient. Root MSE=Root mean squared error. PF= pulmonary fibrosis.
**P<0.001 for all regression models except where denoted by ⁺. **Standardized telomere lengths in quartiles.*

Supplementary Table 10. Stratification by ILD-GAP Score Demonstrates Shorter Mean and Median Leukocyte Telomere Length with Increasing Pulmonary Fibrosis Severity Across Racial/Ethnic groups.

**Characteristics***	Mean Telomere Length (SD)	Median Telomere Length (IQR)
**WHITE (n=1613)**		
0-1	0.04 (0.51)	0.10 (0.63)
2-3	-0.03 (0.50)	-0.01 (0.63)
4-5	-0.11 (0.46)	-0.06 (0.59)
>5	-0.18 (0.44)	-0.20 (0.55)
**BLACK (n=162)**		
0-1	0.38 (0.39)	0.38 (0.54)
2-3	0.26 (0.46)	0.26 (0.57)
4-5	0.17 (0.56)	0.13 (0.71)
>5	-0.18 (0.27)	-0.21 (0.24)
**HISPANIC (n=187)**		
0-1	0.21 (0.50)	0.20 (0.53)
2-3	0.12 (0.51)	0.19 (0.73)
4-5	-0.03 (0.60)	0.02 (0.84)
>5	0.01 (0.37)	-0.15 (0.50)
**ASIAN (n=70)**		
0-1	0.16 (0.61)	0.16 (1.13)
2-3	0.24 (0.54)	0.21 (0.61)
4-5	0.10 (0.30)	0.11 (0.34)
>5	-0.16 (0.52)	-0.17 (0.58)

**Standardized telomere length across study population presented as means (SD). Telomere lengths unadjusted for age or sex. Exception for participants without available components for ILD-GAP score estimation (White n=99, Black n=22, Hispanic n=15, Asian n=3). ILD-GAP=interstitial lung disease, gender, age, physiology [FVC=forced vital capacity, DL_{CO}=diffusing capacity of the lungs] score.*
