# Supplementary material for: Telomere length associates with chronological age and mortality across racially diverse pulmonary fibrosis cohorts
Source: Nat Commun. 2023 Mar 17;14:1489. doi: 10.1038/s41467-023-37193-6 (PMC10023792; doi:10.1038/s41467-023-37193-6)
Supplement: Supplementary file 2 — Reporting Summary [file 41467_2023_37193_MOESM2_ESM.pdf]

nature portfolio

Corresponding author(s): Ayodeji Adegunsoye, MD, MS

Last updated by author(s): 02/08/2023

Reporting Summary

Nature Portfolio wishes to improve the reproducibility of the work that we publish. This form provides structure for consistency and transparency in reporting. For further information on Nature Portfolio policies, see our Editorial Policies and the Editorial Policy Checklist.

Statistics

For all statistical analyses, confirm that the following items are present in the figure legend, table legend, main text, or Methods section.

n/a	Confirmed	
☐	☒	The exact sample size (*n*) for each experimental group/condition, given as a discrete number and unit of measurement
☐	☒	A statement on whether measurements were taken from distinct samples or whether the same sample was measured repeatedly
☐	☒	The statistical test(s) used AND whether they are one- or two-sided<br>*Only common tests should be described solely by name; describe more complex techniques in the Methods section.*
☐	☒	A description of all covariates tested
☐	☒	A description of any assumptions or corrections, such as tests of normality and adjustment for multiple comparisons
☐	☒	A full description of the statistical parameters including central tendency (e.g. means) or other basic estimates (e.g. regression coefficient) AND variation (e.g. standard deviation) or associated estimates of uncertainty (e.g. confidence intervals)
☐	☒	For null hypothesis testing, the test statistic (e.g. *F*, *t*, *r*) with confidence intervals, effect sizes, degrees of freedom and *P* value noted<br>*Give P values as exact values whenever suitable.*
☒	☐	For Bayesian analysis, information on the choice of priors and Markov chain Monte Carlo settings
☒	☐	For hierarchical and complex designs, identification of the appropriate level for tests and full reporting of outcomes
☐	☒	Estimates of effect sizes (e.g. Cohen's *d*, Pearson's *r*), indicating how they were calculated

Our web collection on statistics for biologists contains articles on many of the points above.

Software and code

Policy information about availability of computer code

Data collection	MS-Excel spreadsheet was used in data collection (Microsoft Excel for Mac Version 16.65, 2019)
Data analysis	Stata (2019.R.16; and 2021.R17. StataCorp) and R.v.3.5.1.

For manuscripts utilizing custom algorithms or software that are central to the research but not yet described in published literature, software must be made available to editors and reviewers. We strongly encourage code deposition in a community repository (e.g. GitHub). See the Nature Portfolio guidelines for submitting code & software for further information.

Data

Policy information about availability of data

All manuscripts must include a data availability statement. This statement should provide the following information, where applicable:

- Accession codes, unique identifiers, or web links for publicly available datasets
- A description of any restrictions on data availability
- For clinical datasets or third party data, please ensure that the statement adheres to our policy

The raw telomere length data are protected and are not available due to data privacy laws. The processed telomere length data for the HRS control population are available at "hrsdata" with the identifier [https://hrsdata.isr.umich.edu/data-products/2008-telomere-data]. The pulmonary fibrosis telomere length data that support the findings of this study are not openly available due to reasons of clinical human data sensitivity and are available upon reasonable request from the corresponding author [A.A].

Human research participants

Policy information about studies involving human research participants and Sex and Gender in Research.

Reporting on sex and gender	Reporting Summary: All biological sexes were considered in this observational cohort study and the sex of participants was determined based on self-report as documented within the respective data registries. No consent was obtained for reporting and sharing individual-level data. Results of sex-based analyses which were performed a-priori are reported accordingly.
Population characteristics	The covariate relevant population characteristics are as follows. The mean±SD age was 65±11years for participants with pulmonary fibrosis (PF), and 69±10years for HRS controls. Among participants with PF, the mean standardized leukocyte telomere length (LTL) at PF diagnosis for White (n=1611), Black (n=162), Hispanic (n=183), and Asian (n=70) participants were -0·06 (0·47), 0·37 (0·49), 0·19 (0·54), and -0·04 (0·57), respectively. In the HRS control cohort, the mean standardized LTL for White (n=4319), Black (n=779), Hispanic (n=614), and Asian (n=96), were -0·04 (0·49), 0·08 (0·48), 0·16 (0·51), and 0·19 (0·53), respectively.
Recruitment<br><br>Participants enrolled voluntarily at PF tertiary referral care centers which may have resulted in self-selection of patients with more advanced PF and shorter LTLs than the general population.	All participants were recruited using consensus American Thoracic Society (ATS)/European Respiratory Society (ERS) criteria14,15 and were enrolled after obtaining written informed consent. Study participants were enrolled at diagnosis of PF. Participants enrolled in the Idiopathic Pulmonary Fibrosis Clinical Research Network (IPFnet)16 clinical trials(NCT00650091/NCT00957242) and consented to participate in the optional genetics substudy with available genomic DNA were included in this study. Healthy participants enrolled in the "Health and Retirement Study"(HRS)[NIA-U01AG009740]17 who had available individual-level demographic data and LTL measurements by quantitative polymerase chain reaction (qPCR) assay were included as controls.
Ethics oversight	All relevant hospital institutional review boards approved the study (IRB:UChicago = #14163A; UCSF = #10-01592 & #10-00198; UCDavis = #585448-7 & #875917-2; UTSW = #082010-127 & #AAAS0753).

Note that full information on the approval of the study protocol must also be provided in the manuscript.

Field-specific reporting

Please select the one below that is the best fit for your research. If you are not sure, read the appropriate sections before making your selection.

☒ Life sciences ☐ Behavioural & social sciences ☐ Ecological, evolutionary & environmental sciences

For a reference copy of the document with all sections, see nature.com/documents/nr-reporting-summary-flat.pdf

Life sciences study design

All studies must disclose on these points even when the disclosure is negative.

Sample size	This was an observational cohort study in which all available samples were utilized for the analyses. This is the largest telomere-based study in pulmonary fibrosis to date and is the first study to assess age- and mortality-associations across diverse racial groups in pulmonary fibrosis.
Data exclusions	No data were excluded from the analyses.
Replication	All attempts at replication across the designated racial/ethnic subcategories were successful. Replication was performed five times.
Randomization	Allocation was not random but participants were pre-assigned to self-identified racial categories for the purpose of this analysis.
Blinding	Blinding was not relevant to this study as participants were already preassigned to self-identified racial categories for the purpose of the analysis.

Reporting for specific materials, systems and methods

We require information from authors about some types of materials, experimental systems and methods used in many studies. Here, indicate whether each material, system or method listed is relevant to your study. If you are not sure if a list item applies to your research, read the appropriate section before selecting a response.

Materials & experimental systems

n/a	Involved in the study
☒	☐ Antibodies
☒	☐ Eukaryotic cell lines
☒	☐ Palaeontology and archaeology
☒	☐ Animals and other organisms
☐	☒ Clinical data
☒	☐ Dual use research of concern

Methods

n/a	Involved in the study
☒	☐ ChIP-seq
☒	☐ Flow cytometry
☒	☐ MRI-based neuroimaging

Clinical data

Policy information about clinical studies

All manuscripts should comply with the ICMJE guidelines for publication of clinical research and a completed CONSORT checklist must be included with all submissions.

Clinical trial registration

NCT00470327. (However, this study is not a clinical trial but a multicenter observational cohort study).

Study protocol

Study protocol not available as this study was not a clinical trial but a multicenter observational cohort study.

Data collection

This cohort study used data obtained from patients prospectively enrolled with a confident multidisciplinary diagnosis of fibrotic interstitial lung disease subtypes, collectively referred to as PF, at four tertiary centers: University of Chicago (UChicago), University of California San Francisco (UCSF), University of California Davis (UCDavis), and University of Texas Southwestern, Dallas (UTSW) between September 2003 and December 2019. Healthy participants enrolled in the "Health and Retirement Study" (HRS)[NIA-U01AG009740] who had available individual-level demographic data and LTL measurements by quantitative polymerase chain reaction (qPCR) assay were included as controls.

Outcomes

The primary objective of this study was to investigate whether LTL is associated with chronological age and mortality across racially and ethnically diverse PF cohorts at geographically disparate US sites.

Race and ethnicity data were collected by self-report in prespecified fixed categories, and chronological age was determined at study enrollment. Race and ethnicity comparisons were made between Hispanic participants, non-Hispanic white participants, non-Hispanic Black or African American participants, and non-Hispanic Asian participants (hereafter referred to as Hispanic, White, Black, and Asian participants, respectively). Vital status was determined by reviewing medical records and confirmed using the United States Social Security death index. Lung transplantation status was determined from the EMR of individual sites. Analyses of LTL association with mortality were adjusted for age and sex as these are known to influence mortality in other pulmonary diseases. In all cohorts, mortality refers to all-cause mortality unless otherwise specified.

Telomere Length Analysis: Genomic DNA was isolated from peripheral blood leukocytes obtained at study enrollment. Peripheral blood LTL was measured using qPCR and the RotoGene real-time PCR system (Qiagen) in triplicate, and age-adjusted LTL calculated using normal controls. For comparative analyses LTL was assessed in salivary leukocytes from HRS control subjects. We derived standardized LTL values from the observed (qPCR-based) LTL minus the Cronkhite 2008 expected value and depict these in the results as telomere length (adjusted O-E). LTL measurements are depicted as T/S ratio for all cohorts except for California - where LTL in base pairs (bp) is reported. Given the potential variance in qPCR-based LTL measurement across study sites, standardized LTL values were calculated by applying z-score normalization with multivariable adjustment for age and sex, and categorization into quartiles. This conversion enabled each sample to have the same distribution based on standard deviations empirically computed for each individual across each cohort. In survival analyses, we used LTL below the median (TL50) and transformed TL (negative log-transformed inverse of one minus percentile TL) comparing mortality hazard ratios for each centile of TL to the highest TL centile among subjects with PF.
